# Simple models of quantitative firing phenotypes in hippocampal neurons: Comprehensive coverage of intrinsic diversity

**DOI:** 10.1371/journal.pcbi.1007462

**Published:** 2019-10-28

**Authors:** Siva Venkadesh, Alexander O. Komendantov, Diek W. Wheeler, David J. Hamilton, Giorgio A. Ascoli

**Affiliations:** Center for Neural Informatics, Structures, and Plasticity, Krasnow Institute for Advanced Study, George Mason University, Fairfax, VA, United States of America; Université Paris Descartes, Centre National de la Recherche Scientifique, FRANCE

## Abstract

Patterns of periodic voltage spikes elicited by a neuron help define its dynamical identity. Experimentally recorded spike trains from various neurons show qualitatively distinguishable features such as delayed spiking, spiking with or without frequency adaptation, and intrinsic bursting. Moreover, the input-dependent responses of a neuron not only show different quantitative features, such as higher spike frequency for a stronger input current injection, but can also exhibit qualitatively different responses, such as spiking and bursting under different input conditions, thus forming a complex phenotype of responses. In previous work, the comprehensive knowledge base of hippocampal neuron types *Hippocampome.org* systematically characterized various spike pattern phenotypes experimentally identified from 120 neuron types/subtypes. In this paper, we present a complete set of simple phenomenological models that quantitatively reproduce the diverse and complex phenotypes of hippocampal neurons. In addition to point-neuron models, we created compact multi-compartment models with up to four compartments, which will allow spatial segregation of synaptic integration in network simulations. Electrotonic compartmentalization observed in our compact multi-compartment models is qualitatively consistent with experimental observations. The models were created using an automated pipeline based on evolutionary algorithms. This work maps 120 neuron types/subtypes in the rodent hippocampus to a low-dimensional model space and adds another dimension to the knowledge accumulated in *Hippocampome.org*. Computationally efficient representations of intrinsic dynamics, along with other pieces of knowledge available in *Hippocampome.org*, provide a biologically realistic platform to explore the large-scale interactions of various neuron types at the mesoscopic level.

## Author summary

The neurons in the hippocampus show enormous diversity in their intrinsic activity patterns. A comprehensive characterization of various intrinsic types using a neuronal modeling system is necessary to simulate biologically realistic networks of brain regions. Morphologically detailed neuronal modeling frameworks often limit the scalability of such network simulations due to the specification of hundreds of equations governing each neuron’s intrinsic dynamics. In this work, we have accomplished a comprehensive mapping of experimentally identified intrinsic dynamics in a simple class of models with only two governing equations. We have created over a hundred point-neuron models that reflect the intrinsic differences among the hippocampal neuron types both qualitatively and quantitatively. In addition, we compactly extended our point-neurons to include up to four compartments, which will allow anatomically finer-grained connections among the neurons in a network. Our point-neuron and compact model representations, freely available in *Hippocampome.org*, allow researchers to investigate dynamical interactions among various intrinsic types and emergent integrative properties using scalable, yet biologically realistic network simulations.

## Introduction

Complex interactions among a myriad of neurons make it challenging to study the functions of brain regions. Although each neuron is different, their landmark features such as the dendritic structure and patterns of somatic voltage spikes help define types of neurons, and such grouping allows for a tractable description and investigation of complex network interactions. For instance, large-scale network models of brain regions can include precisely defined neuronal types to create a biologically realistic platform for hypothesis testing. While neurons differ in their morphological, biochemical and electrophysiological features, precisely what features are useful and relevant for neuronal grouping is a topic of great interest [1].

A few studies have created large-scale network models of brain regions [2–5]. The major methodological difference among these studies is the level of biological details captured in the individual components of the network and there is often a tradeoff between such biological details and the scale of the network. For example, a microcircuit model of the rat somatosensory cortex [4] simulated ~31,000 neurons with ~37 million synapses, where each neuron was a biophysically detailed description of one of 207 morpho-electrical types identified experimentally. On the other hand, a large-scale description of thalamocortical systems [2], which used simplified phenomenological neuron models [6], simulated a network of a much larger scale (one million neurons and half a billion synapses), but it only included 22 abstract types among the neurons. Network modeling efforts more specific to the hippocampus include a full-scale model of the CA1 circuit [7] (~338,000 biophysically detailed neuron models of nine types), a large-scale model of the dentate gyrus [8] (~52,000 biophysically detailed neuron models of four types) and a large-scale model of CA1 [9] (~10,000 phenomenological models of two types).

An advantage of biophysically detailed neuron models is that they can include experimentally known distributions of ion channels during model generation. For example, A-type potassium and hyperpolarization-activated currents were distributed non-uniformly (increasing densities with distance from soma) in the CA1 pyramidal models in [7,10] based on experimental observations. However, hundreds of compartments, each specifying the dynamics of several types of currents require prohibitively large supercomputer resources, if one wants to simulate a large-scale network of biophysically detailed neuron models [4,7]. Increasing the complexity of the model also increases the number of free parameters that cannot be measured experimentally and need to be estimated. On the other hand, highly abstract phenomenological models such as [6] specify only two equations, and they significantly reduce the computational cost of simulating large-scale networks [2,9,11]. However, the parameters that govern such models are not directly biologically interpretable and optimizing their parameters to reproduce quantitatively accurate intrinsic dynamics of neuron types can be difficult [12,13]. In current work, with a vision of creating a real-scale network model of the rodent hippocampus that nevertheless captures biological details at the mesoscopic level, we have created phenomenological models of 120 hippocampal neuron types and subtypes using their intrinsic dynamics identified experimentally. Recently, a database of simple models for hundreds of neurons of various transgenic types in the mouse primary visual cortex was created with a similar vision [14].

A large-scale literature mining effort created *Hippocampome.org* [15], a comprehensive knowledgebase of neuron types in the rodent hippocampal formation (dentate gyrus, CA3, CA2, CA1, subiculum, and entorhinal cortex). This resource provides information on morphology, electrophysiology, and molecular marker profiles of more than 100 neuron types, where the type of a neuron is primarily determined based on the locations of its axon, dendrites and soma across 26 parcels of the hippocampus. A numerical protocol [16] was developed to identify the classes of published somatic spike patterns of morphologically identified neuron types. Analysis of a total of 247 traces, which were linked to 90 morphological types, revealed several spike pattern phenotypes, and further divided 22 morphological types into 52 electrophysiological subtypes for a total of 120 neuron types/subtypes. The subtypes of a neuron type, while sharing the same morphological identity, differ in their spike pattern phenotypes. Features of experimentally recorded spike patterns were extracted for a neuron type and a systematic characterization of spike pattern features revealed nine unique families of intrinsic dynamics, such as delayed spiking, non-adapting spiking, simple adapting spiking, and persistent stuttering among hippocampal neurons. Furthermore, many neuron types exhibit different classes of spike patterns for different input currents, resulting in complex spike pattern phenotypes.

In this article, we present a comprehensive set of point neuron models that quantitatively reproduce various spike pattern phenotypes of hippocampal neurons. We also created multi-compartment models that are compact extensions of point neurons in order to allow spatial context for synaptic integration in a network. In addition, our compact multi-compartment (compact-MC) models exhibit electrotonic properties consistent with experimental observations. We also report novel insights into the relationships between abstract model parameters and various biological properties, which were revealed in our correlation analysis. The models were created using an automated modeling framework [13], and they further enhance the existing accumulated knowledge in *Hippocampome.org*, where they are freely available to download. By identifying several possibilities for a quantitative phenotype in phenomenological space, current work comprehensively maps hippocampal neuron types to low-dimensional model subspaces, which can be used as sampling regions for biologically realistic large-scale network simulations of hippocampal circuits.

## Methods

The class of a spike pattern is identified based on various transient and/or steady-state elements present in the pattern. Transient elements are Delay (D), if the first spike latency (*fsl*) is sufficiently long; Adapting Spiking (ASP), if the inter-spike intervals (*ISIs*) increase over time showing a spike frequency adaptation (*sfa*); Rapidly Adapting Spiking (RASP), if a strong *sfa* is only present in the first two or three ISIs, Transient Stuttering (TSTUT), if a quiescent period follows a cluster of high frequency spikes; and Transient Slow-Wave Bursting (TSWB), if a slow after-hyperpolarizing potential follows a cluster of high frequency spikes. Steady-state elements are Silence (SLN), if the post-spike silence (*pss*) (quiescence following the last spike) is sufficiently long; Non-Adapting Spiking (NASP), if no frequency adaptation is identified in a non-interrupted spiking; Persistent Stuttering (PSTUT), if at least one sufficiently long quiescent period separates two clusters of high frequency spikes; and Persistent Slow-Wave Bursting (PSWB) if a slow after-hyperpolarizing potential is present in an otherwise PSTUT pattern. Thus, the key features are *fsl*, *sfa*, *pss* and the number of ISIs (*nISIs*) for a spiking pattern, and burst widths (*bw*), post-burst intervals (*pbi*), number of bursts (*n_bursts*) and *nISIs* within a burst (*b-nISIs*) for a stuttering/bursting pattern.

A spike pattern can consist of one or more elements, and we use a dot (‘.’) notation to separate them. A ‘.’ in a spike pattern indicates that the preceding element is a transient (e.g. “ASP” is a transient element in the pattern “ASP.SLN”), and if the pattern ends with a ‘.’ (e.g. “ASP.” or “RASP.ASP.”), it serves to mean that this is an incomplete pattern, where the duration of current injection was too short to elicit one of the steady state responses. S1 Text and [16] provide more details on the criteria for various spike pattern classes.

The temporal features described above identify the class of a single spike pattern, and all classes of patterns exhibited by a neuron under different input currents collectively define the spike pattern phenotype of that neuron. Thus, our approach emphasizes the temporal patterns in the periodic voltage spikes rather than the shape of the spike or subthreshold dynamics to define the intrinsic dynamics. Note that a minimum of two spikes are required to identify a class, hence, single-spike traces are not assigned a class label in this scheme. However, such single-spike traces are still included as constraints to help capture the excitability (rheobase) in the models more precisely.

We used the Izhikevich model (IM) [6,17] to reproduce spike pattern phenotypes. This model is governed by the state variables membrane voltage (V) and membrane recovery variable (U):

$$C\cdot \frac{dv}{dt}=k\cdot \left(V-V_{r}\right)\cdot \left(V-V_{t}\right)-U+I$$ (1)

$$\frac{dU}{dt}=a\cdot \left\{b\cdot \left(V-V_{r}\right)-U\right\}$$ (2)

$$if\;V=V_{peak}\;then\;V=V_{min},\;U=U+d$$

Where *V*_*r*_ and *V*_*t*_ are the resting and threshold voltages respectively; *V*_*peak*_ is the spike cutoff value, *V*_*min*_ is the post-spike reset value for the voltage and *C* is the cell capacitance. The parameters *K*, *a*, *b* and *d* affect the model’s intrinsic dynamics both qualitatively (e.g. the type of bifurcation revealed by fast-spiking and non-fast-spiking behaviors) and quantitatively (e.g. rheobase and magnitude of *sfa*). Compact-MC models with up to four compartments were modeled using an asymmetric coupling mechanism for the interaction currents in proximal (*I*_*prox*_) and distal (*I*_*dist*_) compartments (e.g. soma and dendrite, respectively) as described in [13]:

$$I_{prox}=G\cdot P\cdot \left(V_{prox}-V_{dist}\right)$$ (3)

$$I_{dist}=G\cdot \left(1-P\right)\cdot \left(V_{dist}-V_{prox}\right)$$ (4)

where *G* is the coupling strength and *P* denotes the degree of coupling asymmetry, which determines the influence of a compartment on the overall model dynamics [18]. It should be noted that each compartment specifies its own set of parameters, except *V*_*r*_ for Eqs (1) and (2). As reported before [13], most of our compact-MC models specify a much weaker coupling toward the soma than away from it, making the somatic compartment dominate the overall model intrinsic dynamics.

Our modeling framework uses evolutionary algorithms (EA) and employs a feature-based error function. By incorporating spike pattern features (*fsl*, *sfa* etc.) and qualitative class criteria (*delay factor*, *number of piecewise linear fit parameters of ISIs* etc.) in the error landscape [16], our approach enforces a fine level of granularity in the key quantitative features of various spike-pattern classes, as described in our previous work [13]. The error function was defined as:

$$error=\;\underset{f\in S}{\sum }\left(W_{f}\times \log\;\left(1+\left|exp_{f}-model_{f}\right|\right)\right)$$ (5)

where *S*: *{fsl, pss, sfa, nISIs}* for a spiking class, and *S*: {*fsl*, *pss*, *bw*, *pbi*, *n*_*bursts*, *b*_*nISIs*} for a bursting/stuttering class. *W*_*f*_ is the feature weight and it was calculated for each feature by comparing the target class with the model spike pattern class during the EA search. The dynamical scaling of errors for the key features using this scheme helped adjust the balance between exploration and exploitation as the population began to converge within the subregion of the target class [13]. Using this scheme, we previously identified subregions for single-behavior neuron types, which show the same class of spike patterns regardless of the input current strength. However, as mentioned before, many neuron types exhibit different classes of patterns under different input currents. We noticed for many such complex phenotypes, the EA with randomly initialized population using broad parameter ranges showed a bias towards a single class rather than reproducing all the classes of a phenotype. To reduce this bias in our current work, the EA population was initialized using the subregions of all desired spike pattern classes, which were identified (in independent EA runs) for single-behavior types [13]. It should be noted, however, that during the EA search, the parameters of the IM could vary beyond these subregions (using an unbounded mutation operator). Furthermore, we used a higher population size (240 as opposed to 120 as for single-behavior types). The operators of the EA (mutation, crossover etc.) were configured by taking into account the features of error landscape created by the IM parameters [19]. In order for a model found by the EA to be accepted, the classes of its spike patterns must match those of experimental traces. There is, however, one exception: without additional dendritic dynamics, the IM failed to reproduce the RASP.ASP. class of patterns, which show a strong and rapid adaptation (in the first 2 or 3 ISIs) followed by a very weak yet sustained adaptation. Therefore, single-compartment models of seven neuron types, which experimentally showed this complex transient pattern, were accepted with RASP.NASP patterns instead (see Results).

Compact-MC models were additionally constrained to exhibit appropriate relative excitabilities and input resistances between soma and dendrites, and sub- and supra-threshold signal propagation properties, as described in our previous work [13]. The following errors were calculated for each dendritic compartment:

$$error_{rheo}=\{\begin{array}{r} 0,II_{dend}^{rheo}\geq I_{soma}^{rheo}\\ \log\;\left(1+\left(I_{soma}^{rheo}-I_{dend}^{rheo}\right)\right),\;II_{dend}^{rheo}<I_{soma}^{rheo} \end{array}$$ (6)

$$error_{vdef}=\{\begin{array}{r} 0,|V_{dend}^{def}\geq V_{soma}^{def}\\ \log\;\left(1+\left(V_{soma}^{def}-V_{dend}^{def}\right)\right),|V_{dend}^{def}<V_{soma}^{def} \end{array}$$ (7)

$$error_{R}=\{\begin{array}{r} 0,\;IR=1\\ \log\;\left(1+\left(1-R\right)\right),\;|R<1 \end{array}$$ (8)

$$error_{epsp}=\{\begin{array}{r} 0,\;0.1\leq EPSP\leq 0.9\\ \log\;\left(1+\left(0.1-EPSP\right)\right),\;EPSP<0.1\\ \log\;\left(1+\left(EPSP-0.9\right)\right),\;EPSP>0.9 \end{array}$$ (9)

where *I*^*rheo*^ is the minimum depolarizing current required to elicit a spike and *V*^*def*^ is the amplitude of steady-state voltage deflection from the resting voltage for a hyperpolarizing input current in decoupled compartments. *R* is the spike propagation rate, defined as the ratio between the number of spikes observed at the destination compartment and the number of spikes initiated at the source compartment. Several AMPA synapses (50–200) were stimulated to initiate a spike at a dendritic compartment and *R* was calculated for the adjacent compartment for forward-spike propagation. Finally, a single AMPA synapse was stimulated at a dendritic compartment and the amplitude of the excitatory post synaptic potential (*EPSP*) was measured at the somatic compartment. A range of (0.1, 0.9) *mV* was enforced for the *EPSP* amplitude. All synapses used a value of 10 for the weight, unless explicitly mentioned otherwise. For the comparison against biophysically detailed models, we used the CA1 Pyramidal multi-compartment model [20] obtained from ModelDB [21] (accession number: 116084), which was simulated using the NEURON simulation environment [22], and the simulated data from [23] for DG Granule neuron type. For the analysis of attenuation of back-propagating spikes, the amplitude of a spike in the IM is measured as the difference between the maximum of *V* and the reset *V*_*min*_. In this article, the goodness-of-fit is reported as the ratio between simulated and experimentally recorded values (for spike pattern features) and the ratio between somatic- and dendritic-compartments (for excitability and input resistance) for intuitive understanding. Optimization and simulation scripts are publicly available at *https*:*//github*.*com/Hippocampome-Org/Time*.

Pairwise correlations were performed to explore the relationships between IM parameters and various pieces of knowledge (PoK) that have been accumulated in Hippocampome.org. All firing pattern classes and electrophysiological properties and the 20 most cited biomarkers were considered, which resulted in a total of 198 correlations. To analyze statistical co-occurrence with existing categorical knowledge, continuous IM parameters were converted into categorical variables appropriately by marking positive and negative or by labelling top- and bottom- one-third ranges respectively as high and low. Correlations between the categorical variables were evaluated using Barnard’s exact test for 2x2 contingency tables. This test provides the greatest statistical power when row and column totals are free to vary [24]. Threshold for statistical significance and false discovery rate for multiple comparisons were conventionally set to 0.05 and 0.25, respectively.

## Results

### Single-compartment models of diverse intrinsic spike pattern phenotypes

**Fig 1:**
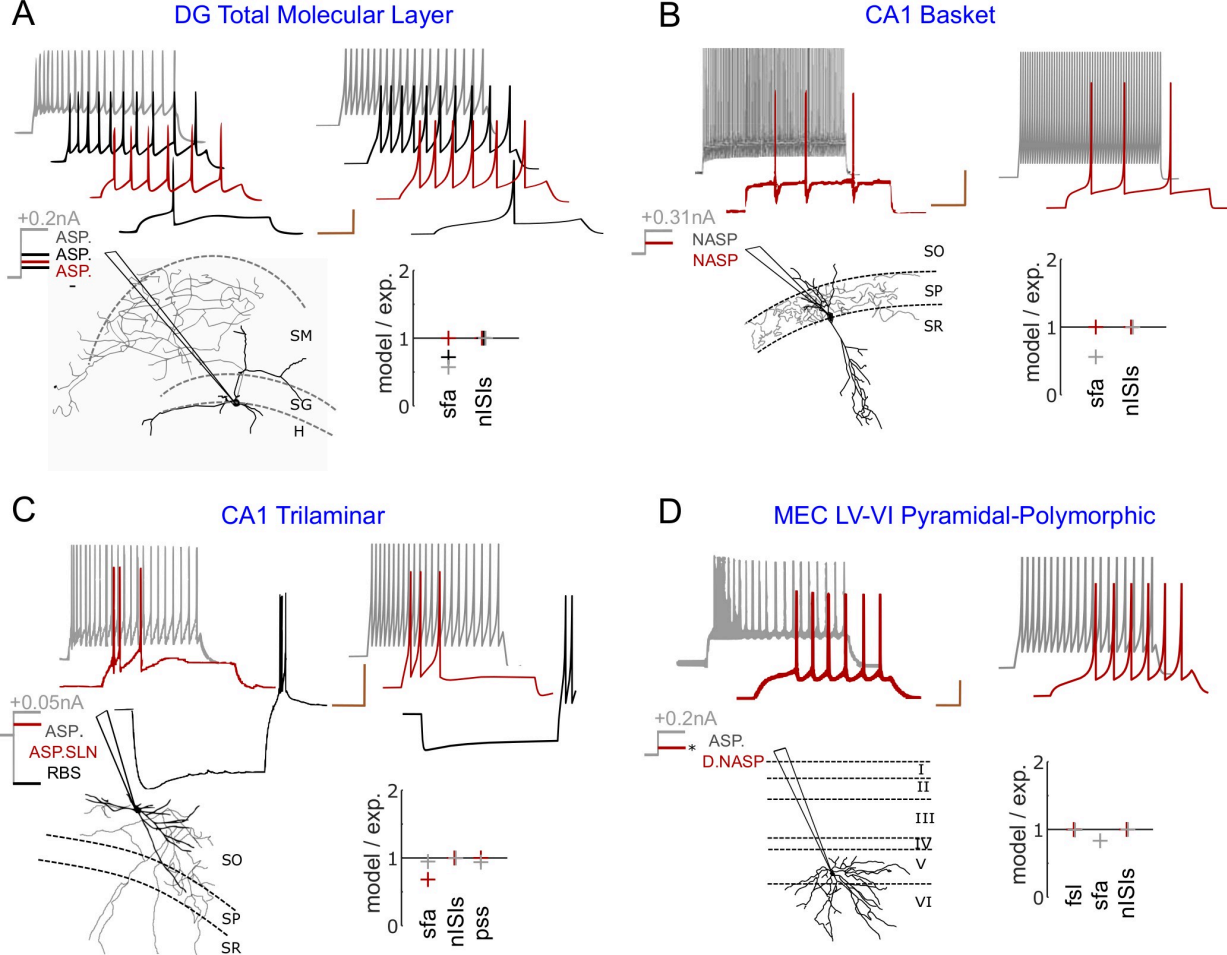
Exemplar models of continuous spiking phenotypes. Each panel displays experimentally recorded voltage traces in the top-left and their morphological identity and magnitudes of somatic current injections in the bottom-left. Traces were digitized by *Hippocampome.org*. Morphological abbreviations: SO—stratum oriens, SP—stratum pyramidale, SR—stratum radiatum, SM—stratum moleculare, SG—stratum granulosum, H–hilus. Model responses for similar input currents (±0.01*nA* from experimental input) are given in the top-right and the goodness-of-fit is given (only) for key features in the bottom-right (S2 Table reports goodness-of-fit for all features). The traces are highlighted in different colors to visually compare experimental and model responses, and to identify the input current and key features for each trace. Calibration bars denote 200*ms* and 20*mV* in all panels. (**A**) Simple phenotype of a dentate gyrus (DG) Total Molecular Layer neuron that elicits ASP. patterns under three different input currents [25]. The digitally reconstructed morphology was reproduced from *NeuroMorpho.Org* [26]. (**B**) Simple phenotype of a CA1 Basket neuron that elicits patterns of class NASP for +0.15*nA* and +0.31*nA* [27]. Note that *sfa* in the red trace is not statistically significant to qualify this pattern as ASP. (**C**) The phenotype of a CA1 Trilaminar neuron shows different classes of patterns for +0.025*nA* and +0.05*nA* [27]. In addition, this neuron elicits rebound spikes (RBS) for a hyperpolarizing input of -0.1*nA*. (**D**) The phenotype of a medial-entorhinal cortex (MEC) neuron shows different classes of patterns for +0.2*nA* and an unknown input (denoted by ‘*’) just above its rheobase [28]. All experimental traces are whole-cell patch clamp recordings.

**Fig 2:**
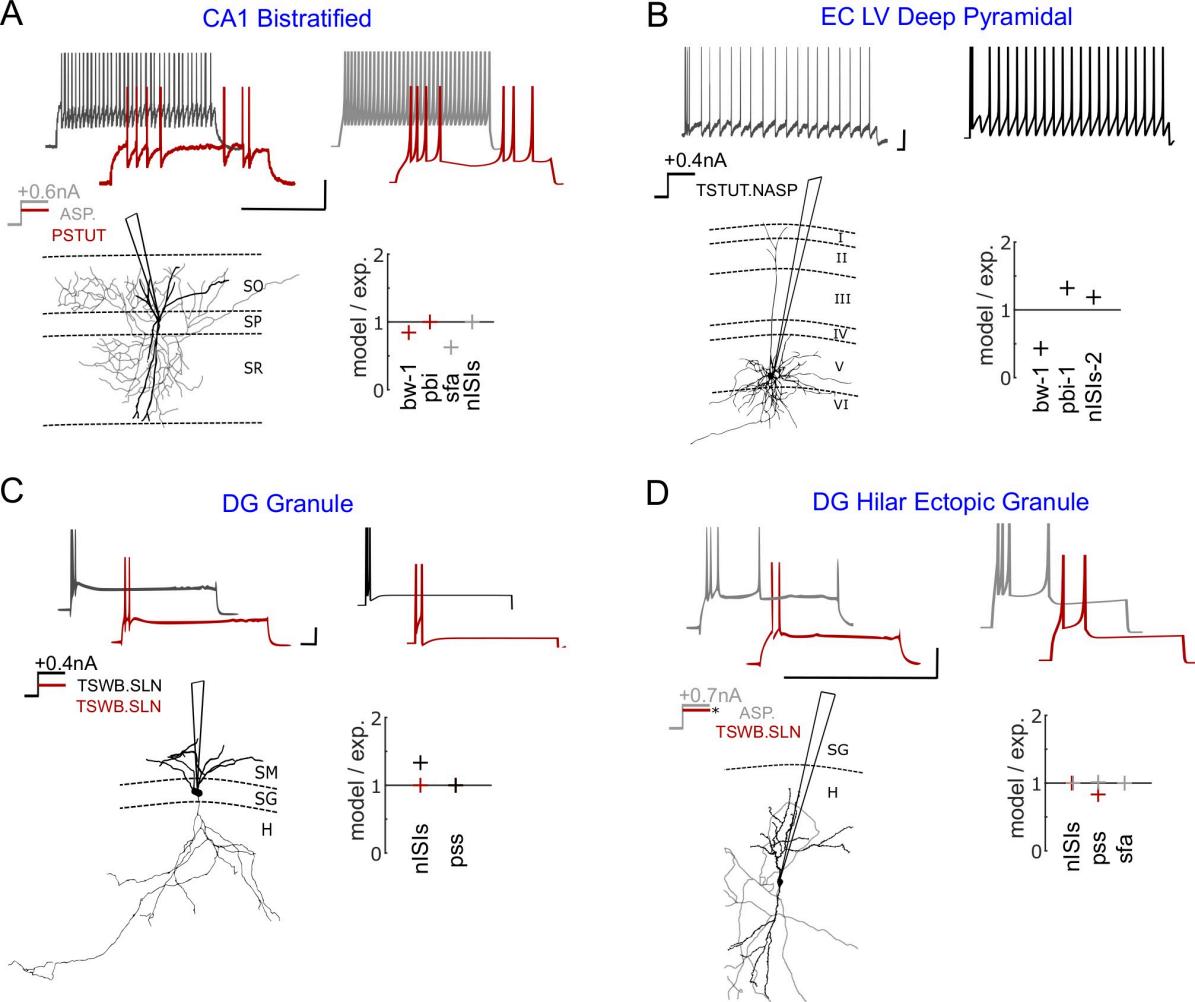
Exemplar models of stuttering/bursting phenotypes. (**A**) Complex phenotype of a Bistratified neuron in CA1. This neuron elicits a stuttering pattern for +0.4*nA* (red) and a spiking pattern for +0.6*nA* (grey) [29]. The digitally reconstructed morphology [30] was reproduced from *NeuroMorpho.Org* [26]. (**B**) The voltage trace recorded from an entorhinal layer-5 neuron shows both (transient) bursting and (steady state) spiking features for +0.4*nA* [31]. (**C**) A DG granule neuron transiently bursts for both +0.2*nA* and +0.4*nA* with quantitative difference [32]. The digitally reconstructed morphology was reproduced from *NeuroMorpho.Org* [26,33]. (**D**) A dentate gyrus neuron that transiently bursts just above its rheobase (red) elicits a spiking pattern with a strong *sfa* (grey) for a higher input current [34]. ‘*’ indicates the unknown magnitude of the input current near rheobase. All voltage traces were digitized by *Hippocampome.org*. Experimental spike amplitudes are truncated. Calibration bars denote 200*ms* and 20*mV* in all panels. Goodness-of-fit is given only for key features (see S3 Table for all features). The experimental traces in panels A, B and D are intracellular recordings and the trace in panel C is a whole-cell patch clamp recording.

The intrinsic dynamics of a neuron is typically identified in experiments by injecting step input currents of various magnitudes. A neuron’s responses to these inputs typically fall into one of two phenotype super-families: (1) a spiking phenotype, where the neuron only exhibits continuous spike pattern classes such as ASP.SLN, NASP, and D.NASP for different input currents (Fig 1A–1D), and (2) a stuttering/bursting phenotype, where the neuron exhibits an interrupted spike pattern class such as TSWB.SLN, TSTUT.NASP, and PSTUT for at least one input current (Fig 2A–2D). A spiking or stuttering phenotype could be formed by various combinations of spike pattern classes, and models for four exemplar cases in each of these two phenotype super-families are reported in this article (visit *Hippocampome.org* for a comprehensive list of phenotypes and their models).

In the simplest case, a neuron exhibits spike patterns of the same class regardless of the input current strength. For example, the three spike patterns recorded under different input currents from a DG Total Molecular Layer neuron were identified as ASP. (Fig 1A), and the two patterns recorded from a CA1 Basket neuron were identified as NASP. (Fig 1B). Such simple-behavior neurons typically show different quantitative features among different patterns of the same class. In the former example, the three ASP. traces were experimentally recorded under +0.075*nA* (red), +0.100*nA* (black), and +0.200*nA* (grey) [25]. The ISI counts (*nISIs*) are 5, 9, and 19, and *sfa* magnitudes are 0.142, 0.114, and 0.056 respectively for the red, black and grey traces. The model of this neuron type was constrained to quantitatively reproduce the spike pattern features for similar input currents: *nISIs* of 5, 9 and 19, and sfa magnitudes of 0.142, 0.082, and 0.032 respectively for +0.073*nA*, +0.102*nA* and +0.205*nA*. S1 Fig illustrates examples of models reproducing frequency responses of neurons for a range of input currents.

Additionally, a neuron can show more complex behaviors by eliciting patterns of different classes under different input currents (Fig 1C and 1D). Both CA1 Trilaminar and MEC LV-VI Pyramidal-Polymorphic neurons include ASP. in their phenotypes (grey traces), but they show different dynamics close to their respective rheobases. Whereas the former quickly fires a few spikes before going into a silence mode (ASP.SLN), the latter shows delayed-spiking (D.NASP). The model quantitatively reproduces the characterizing features of these different classes (see *pss* for ASP.SLN and *fsl* for D.NASP). Also, note that the model reproduces the rebound-spiking behavior for a hyperpolarizing input current, a known feature of CA1 Trilaminar neurons [27].

Another level of complexity in spike pattern phenotypes is when the intrinsic dynamics show sharply distinguishable spike-pattern classes, which differ across super-families, under different input conditions. For example, a CA1 Bistratified neuron stutters (PSTUT) for +0.4*nA*, and spikes for +0.6*nA* (ASP.) (Fig 2A). A few neuron types and subtypes in the hippocampus exhibit such a complex phenotype, where PSTUT is typically observed just above the rheobase of a neuron (e.g. CA1 Neurogliaform [35] and DG Total Molecular Layer subtype [25]). Our simple models capture the characterizing features of both PSTUT and ASP. (Fig 2A) under the right input conditions. It is worth mentioning that all PSTUT neurons are inhibitory neurons and the CA1 region has a proportionately larger number of these phenotypes [16,36]. In many cases, however, the characteristic features of interrupted spiking can be only transiently present (Fig 2B). Here, a single pattern presents features of both bursting and spiking, where a relatively longer interval separates a few high frequency spikes (burst) from a train of regular spikes. In another set of examples, Granule and Hilar Ectopic Granule cells in the dentate gyrus (DG) show only transient bursting just above their respective rheobases (Fig 2C and 2D). However, for an increased input current, Granule cells still maintained the same TSWB.SLN pattern with quantitative differences such as increased number of spikes, whereas Hilar Ectopic Granule cells transitioned to ASP. These constrained representations of two different DG neurons fall under the same family of non-persistent bursting, but they are optimized to capture the finer quantitative differences in the input-dependent responses between these two neuron types. Thus, our simple models do not only qualitatively capture the rich diversity of dynamical classes defined systematically, but they are also quantitatively constrained representations of experimentally recorded patterns from hippocampal neuron types.

### Multi-compartment models as compact extensions of point-neuron models

The point-neurons with only two state variables, which were presented in the last section, would tremendously reduce the computational cost of simulating large-scale networks of hippocampal circuits relative to morphologically detailed Hodgkin-Huxley type models. However, since they lack spatial dimension, they do not differentiate synaptic inputs from different layers, unlike their biological counterparts. For example, hippocampal pyramidal neurons receive entorhinal projections on the apical dendrites in stratum lacunosum moleculare (SLM), and intra-hippocampal connections in stratum radiatum (SR), thereby compartmentalizing synaptic integration of distinct laminar inputs. While it is not possible to spatially segregate synaptic integration in a network of point-neurons, it is of interest to see the effects of such segregated synaptic integration mechanisms in a network. *Hippocampome.org* (version 1.4) identifies 87 neuron types with their dendrites invading at least two layers. Therefore, for these neuron types, in addition to point neuron models, we created compact-MC models with up to four compartments. Here, each compartment corresponds to a hippocampal layer, allowing layer-level connectivity specifications at the neuron type level.

**Fig 3:**
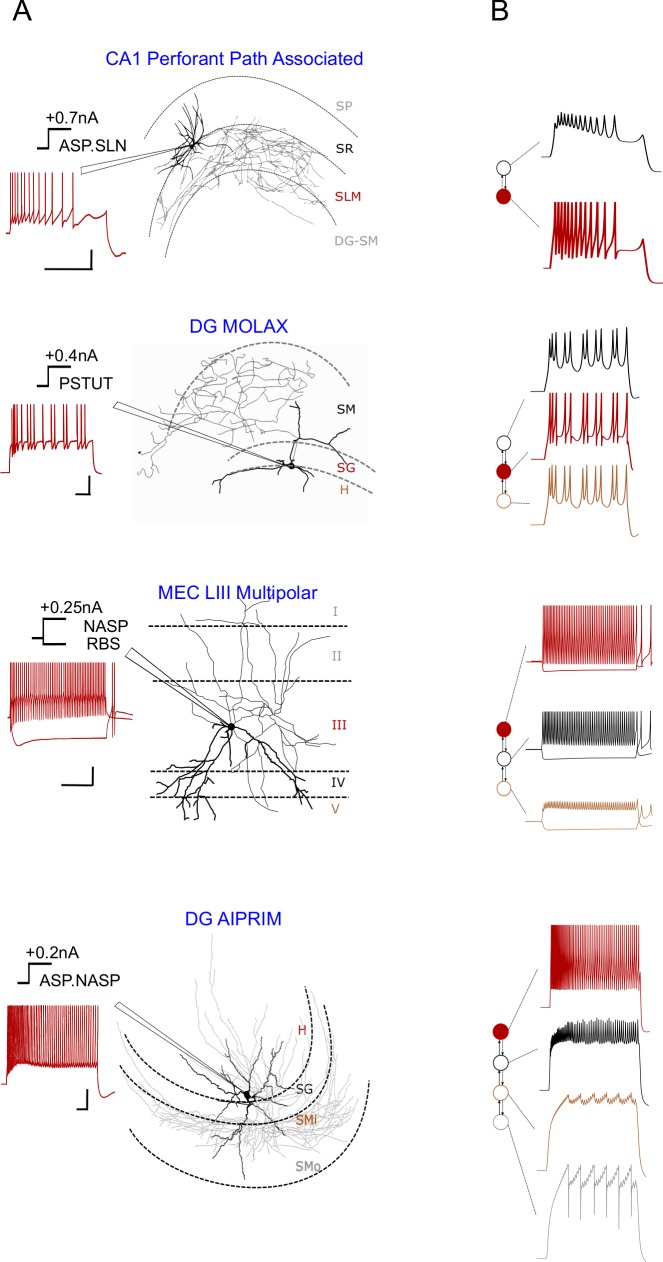
Multi-compartment models compactly extend point neurons to allow layer-level spatial context. (**A**) Experimentally recorded somatic voltage traces (left) are given for four different morphological types (right). Dendritic invasion (darker) of layers and relative soma location determine the number and layout of compartments. (**B**) Layout of compartments coupled asymmetrically (left) correspond to the layers of dendritic invasion shown in A. Filled circles denote somata. Compartment responses for somatic input currents that are ±0.01*nA* from experimental input are shown on the right side. See Fig 4 for quantitative comparison of spike pattern features, S2 Fig for dendritic features, and Fig 5 for another possible 4-compartment layout. The digitally reconstructed morphology of DG MOLAX [25] was reproduced from *NeuroMorpho.Org* [26]. Other experimental traces were digitized by *Hippocampome.org* from the following sources (from top to bottom): [39], [25], [28] and [40]. Morphological abbreviations: SMi and SMo–inner one-third and outer two-third of stratum moleculare. Experimental spike amplitudes are truncated. Calibration bars denote 200*ms*, 20*mV*. The experimental trace ASP.SLN is an intracellular recording and the remaining experimental traces are whole-cell patch clamp recordings.

**Fig 4:**
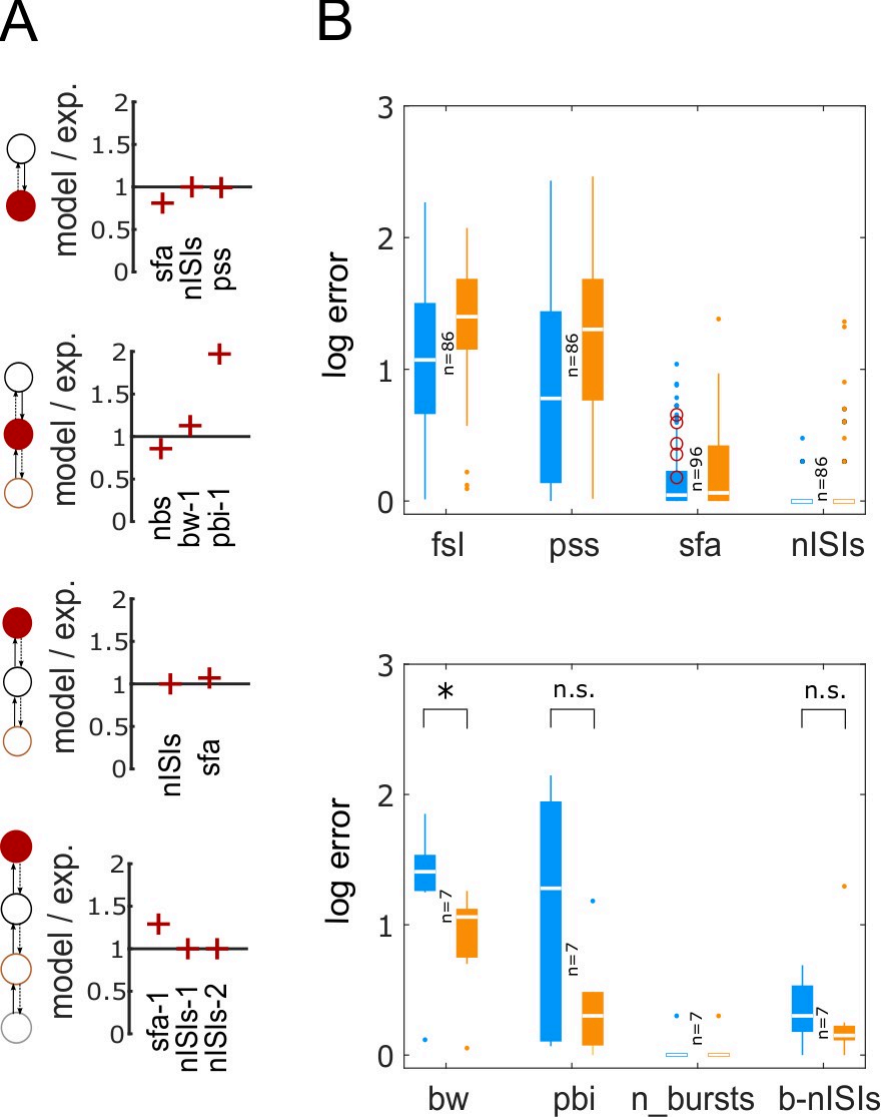
Accuracy of compact multi-compartment models in reproducing spike pattern features. (**A**) The goodness-of-fit is given (only) for key features for each of the four examples from Fig 3 (see S4 Table for all features). (**B**) Pairwise comparisons of accuracy between single-compartment (blue) and compact-MC (orange) models for spiking features (top) and bursting features (bottom). While single-compartment models, in general, showed smaller errors for spiking features, they did not satisfy statistical criteria for RASP.ASP. patterns (denoted by circles in top panel). See S4 Fig for an example for RASP.ASP. pattern. At the same time, compact-MC models generally improved the accuracy of bursting features (bottom) with a significant improvement in *bw* (p<0.005 for paired-sample *t-*test).

One example for each of the four out of five possible multi-compartment layouts are illustrated here, and the fifth layout is discussed in detail in Section 3.3. The somatic compartment of a compact-MC model quantitatively reproduces the spike patterns experimentally recorded from the soma of the respective neuron type for similar input currents (Figs 3 and 4A). The number and layout of the coupled compartments is determined by the layers of dendritic invasion and known/possible soma locations of real neurons as illustrated by various examples in Fig 3. The dendritic compartments in a compact-MC model are less excitable and have higher input resistances than the somatic compartment (S2B Fig) [37,38].

Furthermore, forward-coupling (from dendrite to soma) between compartments is just strong enough to evoke a somatic excitatory postsynaptic potential (EPSP) with an amplitude in the range [0.1, 0.9] *mV* for a single synaptic stimulation at a dendritic compartment and to achieve a forward-spike propagation (from dendrite to soma) ratio in the range [0.5, 1.0] (S2C and S2D Fig). Forward- and backward-coupling strengths are defined by *G*·*P* and *G*·(1−*P*) respectively (see Eqs 3 – 4). As mentioned in Methods, the backward-coupling (from soma to dendrite) is much stronger than the forward-coupling in most of our compact-MC models, consistent with the electrotonic profiles reported for various neuron types [41–43]. Such an asymmetric design for coupling enables the somatic compartment to dominantly define the model’s overall intrinsic dynamics, while still preserving forward propagation properties for sub- and supra-threshold signals from dendrites. Thus, our multi-compartment models are compact extensions of point neuron models, which allow spatial contexts for synaptic integration.

Although the major motivation for creating compact-MC models is to allow synaptic segregation in a network model, we also investigated if additional dendritic mechanisms implemented in our compact-MC models could help achieve a better fitting of somatic spike patterns than their point-neuron counterparts. Therefore, we performed pairwise comparisons between the somatic spike pattern features of single-compartment and compact-MC models. In general, implementing additional dendritic mechanisms in the models only improved the accuracy of bursting features (Fig 4B). Interestingly, *fsl* and *pss* errors were higher in the models due to the addition of dendritic compartments. However, it should be noted that each additional compartment not only adds two state variables, which require more computations for numerical simulation, but also adds ten open parameters (including coupling parameters) making it a more-challenging optimization task. Although our single-compartment models were able to reproduce quantitatively comparable experimental bursting/stuttering patterns (Fig 2) (see [13] for two exceptions), compact-MC models significantly improved the accuracy of *bw*, a key feature of bursting/stuttering patterns (Fig 4B).

Furthermore, while the single-compartment models quantitatively captured various classes of adapting spike pattern phenotype such as ASP., ASP.SLN, ASP.NASP and RASP.NASP, they failed to reproduce RASP.ASP. patterns. These patterns exhibit a strong and rapid adaptation in the first few ISIs, which is then followed by a very weak and sustained adaptation. Interestingly, we found that such a combination was not possible in the IM (red circles in Fig 4B), unless additional dendritic compartments were included. Two different time constants (parameter ‘*a*’) for the adaptation variable (state variable *U*) were required for the somatic and dendritic compartments, respectively, in order to capture such complex transients in the soma. In our single-compartment models, RASP.ASP. is represented by RASP.NASP, since the adaptation followed by RASP. is usually very weak. See S4 Fig for an example.

### Properties of dendritic compartments in compact-MC models

**Fig 5:**
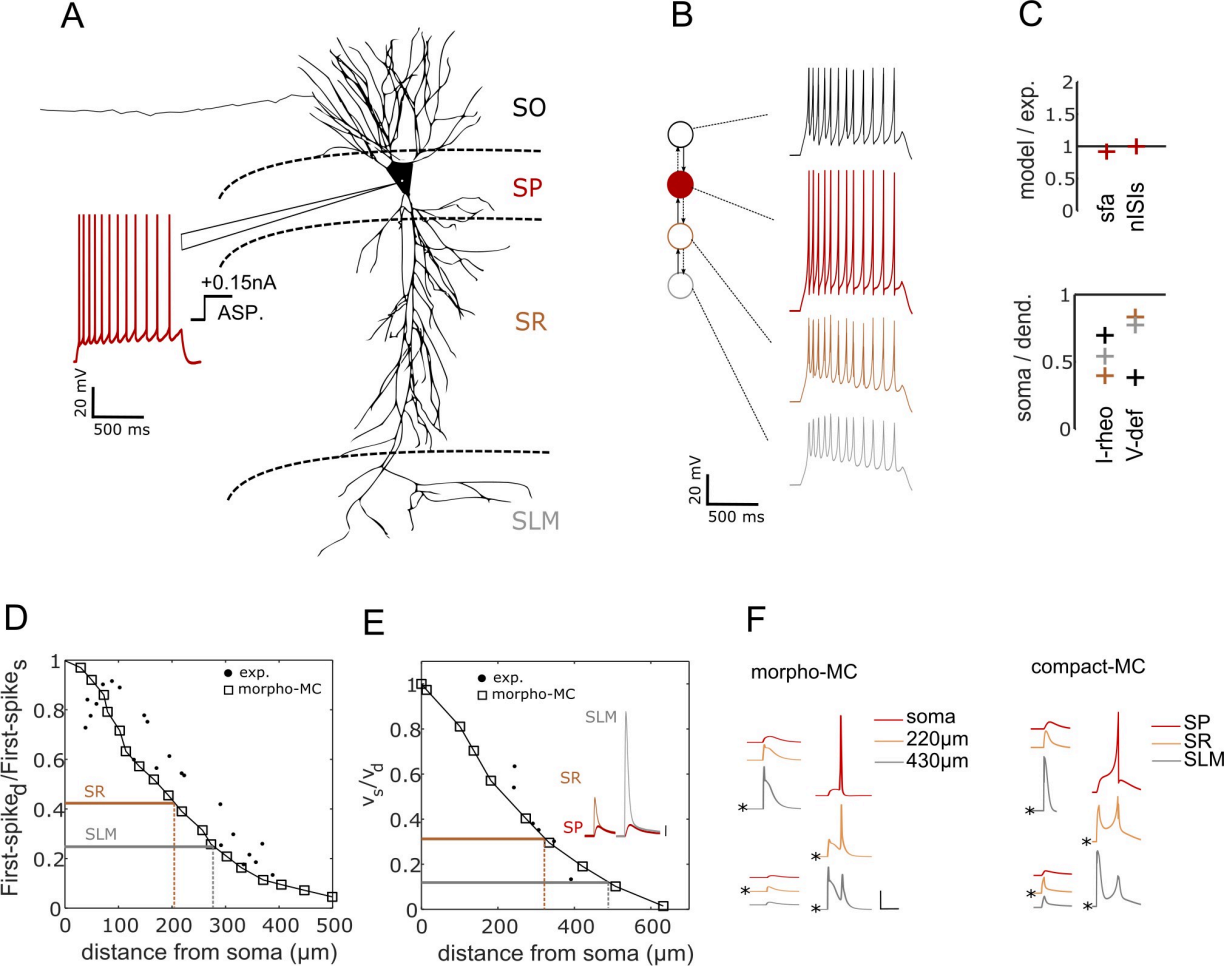
A 4-compartment model of CA1 Pyramidal neuron. (**A**) A whole-cell patch clamp recording from a CA1 Pyramidal neuron [44] digitized by *Hippocampome.org*, and digitally reconstructed morphology of the same type [45] reproduced from *NeuroMorpho.Org* [26]. (**B**) Layout of the compartments (left) and their responses to somatic input current of +0.15*nA*. (**C**) Somatic compartment reproduces key features that are quantitatively comparable to the experimental features (top). Minimum depolarizing input required to elicit a spike (I-rheo) and steady-state voltage deflection (V-def) for a hyperpolarizing input are higher in dendritic compartments than the somatic compartment (bottom). (**D**) Attenuation of first back-propagating spike from experimental recordings (exp.), in morphologically detailed multi-compartment model (morpho-MC) and in compact-MC model (horizontal lines; for the spike trains in B). First-spike_d_ and First-spike_s_ denote amplitudes of first spikes at dendrite and soma, respectively. Vertical lines indicate the distances of the morpho-MC sections from the soma that correspond to the attenuation profiles of proximal (SR) and distal (SLM) compartments in the IM. (**E**) Attenuation of dendritic EPSPs as they propagate towards the soma. V_s_ and V_d_ are amplitudes of EPSPs at soma and dendrite, respectively. Inset shows compartment responses for a single synaptic stimulation at SR (left) and SLM (right). Calibration: 0.2mV. (**F**) Conditional spike propagation in morpho-MC (left) and compact-MC (right) models of a CA1 Pyramidal neuron. ‘*’ denote the stimulated compartment. A single spike initiated at the distal compartment (top left traces: stimulation at 430μm in SLM) failed to propagate to the soma. Additional depolarization level at the proximal compartment (bottom left traces: stimulation at 220μm in SR) facilitates propagation of spike initiated at the distal compartment (right traces) to the soma in both models. Experimental data digitized from [46] (D) and [47](E). Multi-compartment CA1 Pyramidal model from [20] was used to obtain morpho-MC data. Calibration: 20mV, 20ms.

In addition to the features discussed in the last section, our compact-MC models show electrotonic structures and interplay between different compartments that are similar to those observed experimentally and in morphologically detailed multi-compartment (morpho-MC) models. To illustrate this, here we present a 4-compartment model of CA1 Pyramidal neurons and discuss the spike propagation and voltage attenuation properties of apical compartments. First of all, the somatic compartment quantitatively captures frequency adaptation (Fig 5B), a characterizing feature of experimentally recorded spike patterns from CA1 Pyramidal neurons (Fig 5A [44] and Fig 5C—top). Secondly, the dendritic compartments (SR, SLM and SO) are less excitable and have higher input resistances than the somatic compartment (Fig 5C—bottom).

CA1 Pyramidal neurons exhibit strong attenuation of spike amplitudes as they propagate from the soma to apical dendrites [46,48]. This attenuation has been attributed to the highly dense expression of transient A-type K^+^ conductance in the dendrites [48,49]. We compared the attenuation of back-propagating spikes between compact-MC and morpho-MC models (Figs 5D and S3A). The compartments SR and SLM in the compact-MC model matched the attenuation profiles of morpho-MC sections at ~210μm and ~275μm, respectively, from the soma. However, it should be noted that the experimental dendritic recordings distal to ~300 μm showed a dichotomy of attenuation exhibiting either strong (71–87%) or weak (26–42%) attenuation [46], and we only included data corresponding to strong attenuation for comparison in Fig 5D.

In real neurons, integration of an EPSP is influenced by the location of the synapse, because the voltage attenuates more from a distal dendritic location to the soma, than from a proximal location. This is partly due to the higher input resistances of more distal dendrites with smaller diameters. Such an attenuation profile will also hold for a uniform diameter cable with appropriate electrotonic asymmetry between the ends. The attenuation of EPSPs in our compact-MC models is consistent with experimental observations and morpho-MC simulations (Figs 5E and S3B). The SR and SLM compartments in the compact-MC model matched the EPSP attenuation profiles of morpho-MC sections at ~320μm and ~470μm from the soma, respectively. It has been shown in some CA1 Pyramidal neurons that the synapses might be able to compensate for their distance by scaling their conductances in order to sufficiently influence somatic voltage [50,51]. In our model, compared to a synapse stimulated at SR to evoke a somatic (SP) EPSP with an amplitude of 0.2*mV*, a 4-fold increase in synaptic weight was required at SLM in order to evoke an EPSP with the same amplitude at SP (Fig 5E inset).

Furthermore, the distal compartments in our 3- and 4-compartment models rarely initiated a spike that successfully propagated to the soma, and additional depolarization levels at the proximal compartment facilitated forward-propagation of spikes from distal compartments. Such an interplay between proximal and distal compartments in a compact-MC model is qualitatively comparable to that of a morpho-MC model (Fig 5F). This is also consistent with the experimental observation that the activation of CA1 neurons by perforant path alone, which projects to SLM, is limited, but modest activation of Schaffer-collateral synapses in SR facilitates forward propagation of distal spikes [20]. It has thus been suggested that Schaffer-collateral evoked EPSPs “gate” perforant path spikes in CA1 Pyramidal neurons, underscoring the functional interaction between these different dendritic domains [37]. Such compartmentalization might be crucial to appropriately capture the integration of distinct laminar inputs and it is therefore notable that our compact-MC models with “active” dendritic compartments qualitatively reproduce such observations. Although voltage attenuation profiles could be modeled in IMs with passive dendritic compartments that are appropriately coupled, capturing the interplay between compartments such as gating as demonstrated here requires that the distal dendrites are optimized to initiate a spike.

**Fig 6:**
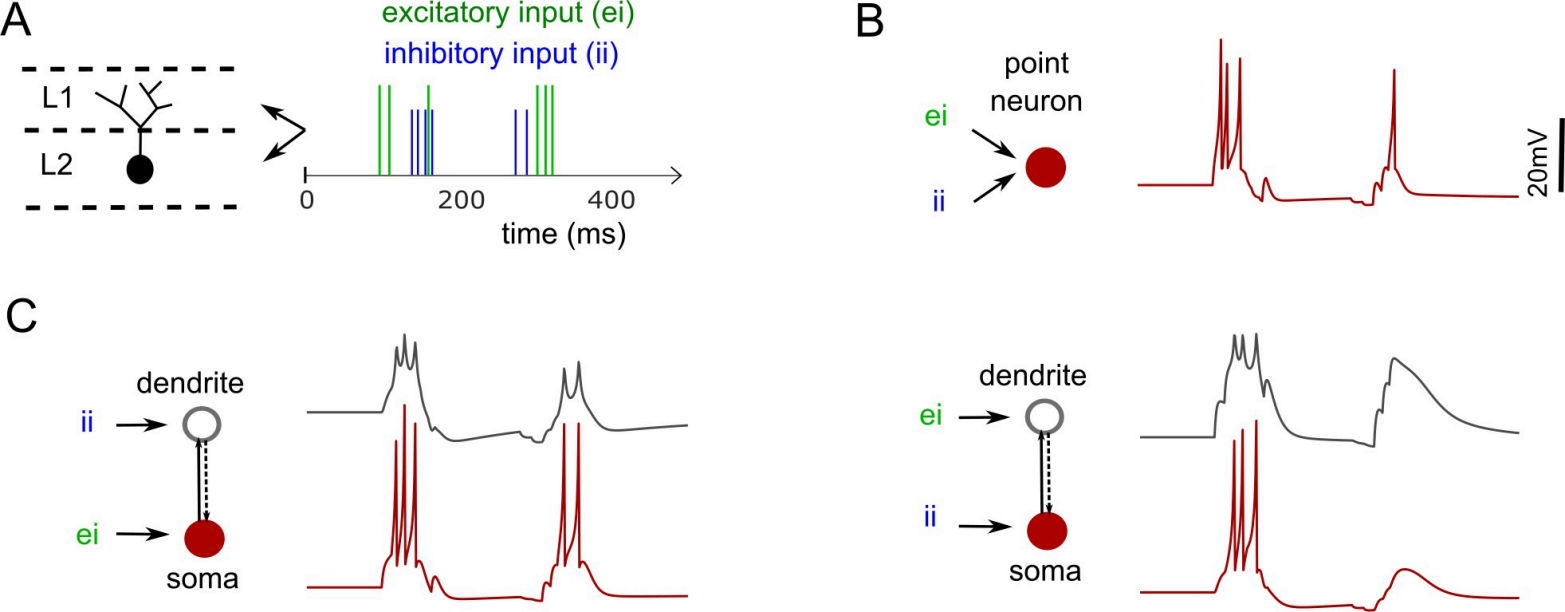
An example of segregated synaptic integration in a 2-compartment model. (**A**) A schematic illustration of a biological neuron with its soma and dendrites in different layers L1 and L2. This neuron receives two distinct presynaptic spike trains in L1 and L2. (**B**) A simplified point-neuron model integrates both the excitatory and inhibitory presynaptic spikes at the same point. (**C**) A 2-compartment model (see Fig 3) can integrate distinct inputs in different compartments. The model behaves differently (left vs. right) depending on the location of integration of distinct inputs. Excitatory weight = 8.0 and Inhibitory weight = 1.5 for B and C, respectively. Dendritic synapses scale 4x (see Fig 5E inset for details).

Finally, spatially segregating the temporal integration of presynaptic spikes might enhance the range of responses of the postsynaptic neuron. We illustrate this using a simple example, where we compared the responses of single- (point neuron) and 2-compartment models of a CA1 Perforant Path-Associated neuron (from Fig 3) for arbitrary excitatory and inhibitory presynaptic spike trains (Fig 6). Following a spike-triplet, while the point neuron elicited a single spike, the 2-compartment counterpart exhibited different responses (2 or 0 spikes) depending on the location of integration of distinct spike trains. To what extent these differences influence the emergent network properties remains to be answered, but our models allow one to explore such questions.

### Online repository of models: An enhancement to *Hippocampome.org*

A comprehensive list of models of 68 types and 52 subtypes of neurons is freely available at *Hippocampome.org*. Mapping the intrinsic dynamics of each neuron type in a low-dimensional model space enhances the existing information accumulated in this rich knowledge base of hippocampal neuron types.

**Fig 7:**
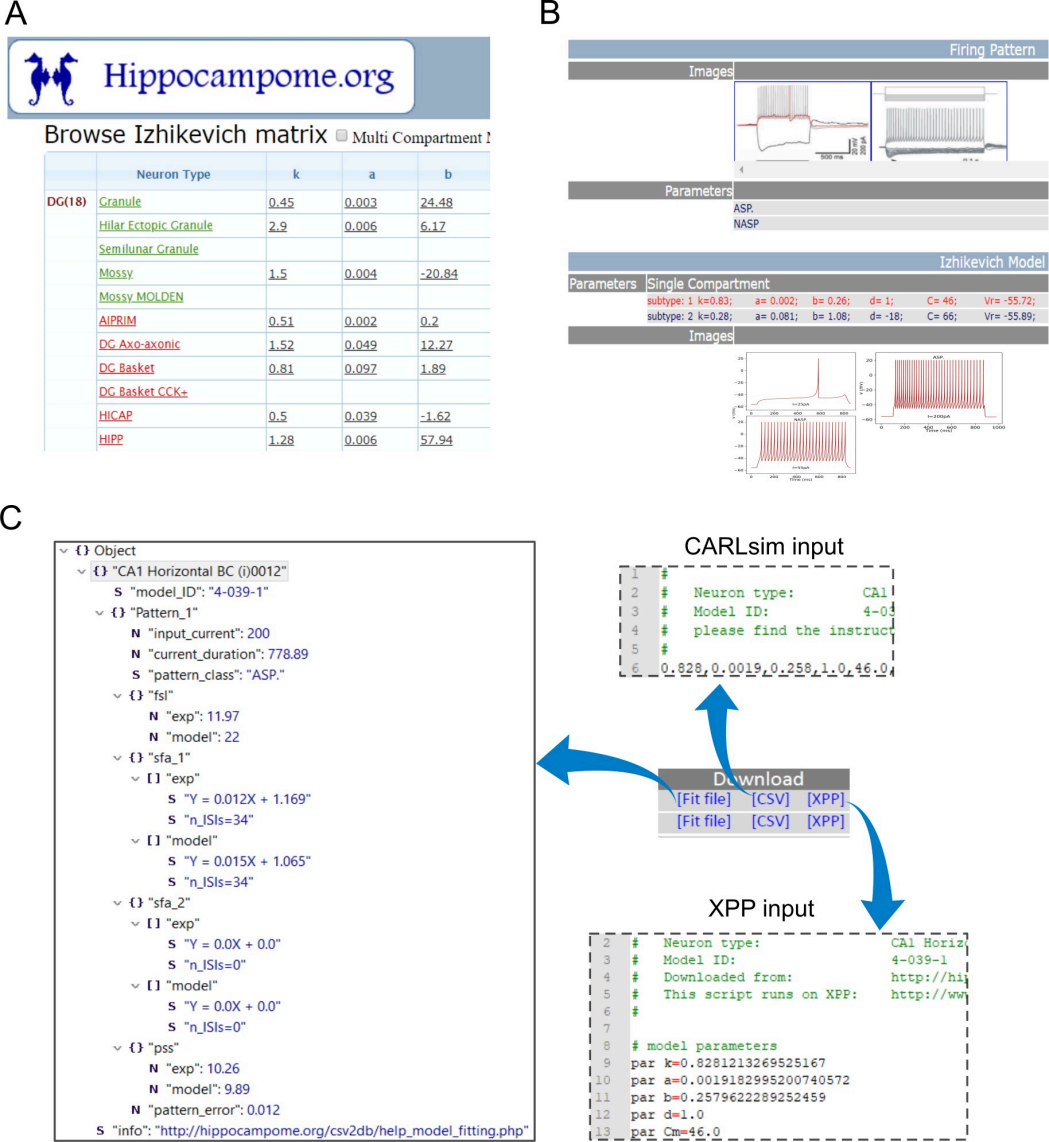
*Hippocampome.org* provides a comprehensive list of models and ready-to-run scripts. **(A)** Single- and multi-compartment model parameters for all neuron types are presented in a matrix form on the main page. Each row is linked to a Neuron page. (**B**) The Neuron page for each neuron type has been enhanced to include model parameters and simulated traces for all types and subtypes (if any). (**C**) The Neuron page provides the user with three downloadable files for each subtype: a *Fit-file* that lists both experimental and simulated features for each pattern, an XPP script to simulate single-compartment models, and a CARLsim input file for single- and multi-compartment models.

All the single-compartment and compact-MC model parameters are presented in a matrix on the main page for easy browsing (Fig 7A). Within a Neuron page, models for all subtypes (if any) of the given morphological type are available for download. This page includes both the experimentally recorded voltage traces and simulated ones for all models (Fig 7B). Simulated spike patterns are also annotated with their class labels. Each type/subtype presents three downloadable files (Fig 7C): a *Fit-file* including both the experimental and simulated values for spike pattern features such as *fsl* and *sfa* for each available pattern in a JSON format; an XPP [52] script for single-compartment models; and a csv input file that includes both single-compartment and compact-MC models for CARLsim [11], a high performance GPU-based simulator. The Help section of *Hippocampome.org* provides links to explanatory pages on model definition, fitting, and simulation, including instructions to run the scripts and feature descriptions, under “Simulation of Firing patterns”. This section also provides a link to download all single-compartment model descriptions in simulator-independent NeuroML format [53]. Furthermore, neuron pages include an online simulator, which allows the simulation of single-compartment dynamics for custom input.

### Relationship between model parameters and biological features

A limitation of many phenomenological models such as the IM used here is the lack of biological interpretability of their parameters. One advantage of our approach of densely covering the diversity among neuron types is to allow one to explore relationships between the mathematical parameters of the IM and various known biological features. Our analysis revealed interesting trends and correlations in this regard.

**Fig 8:**
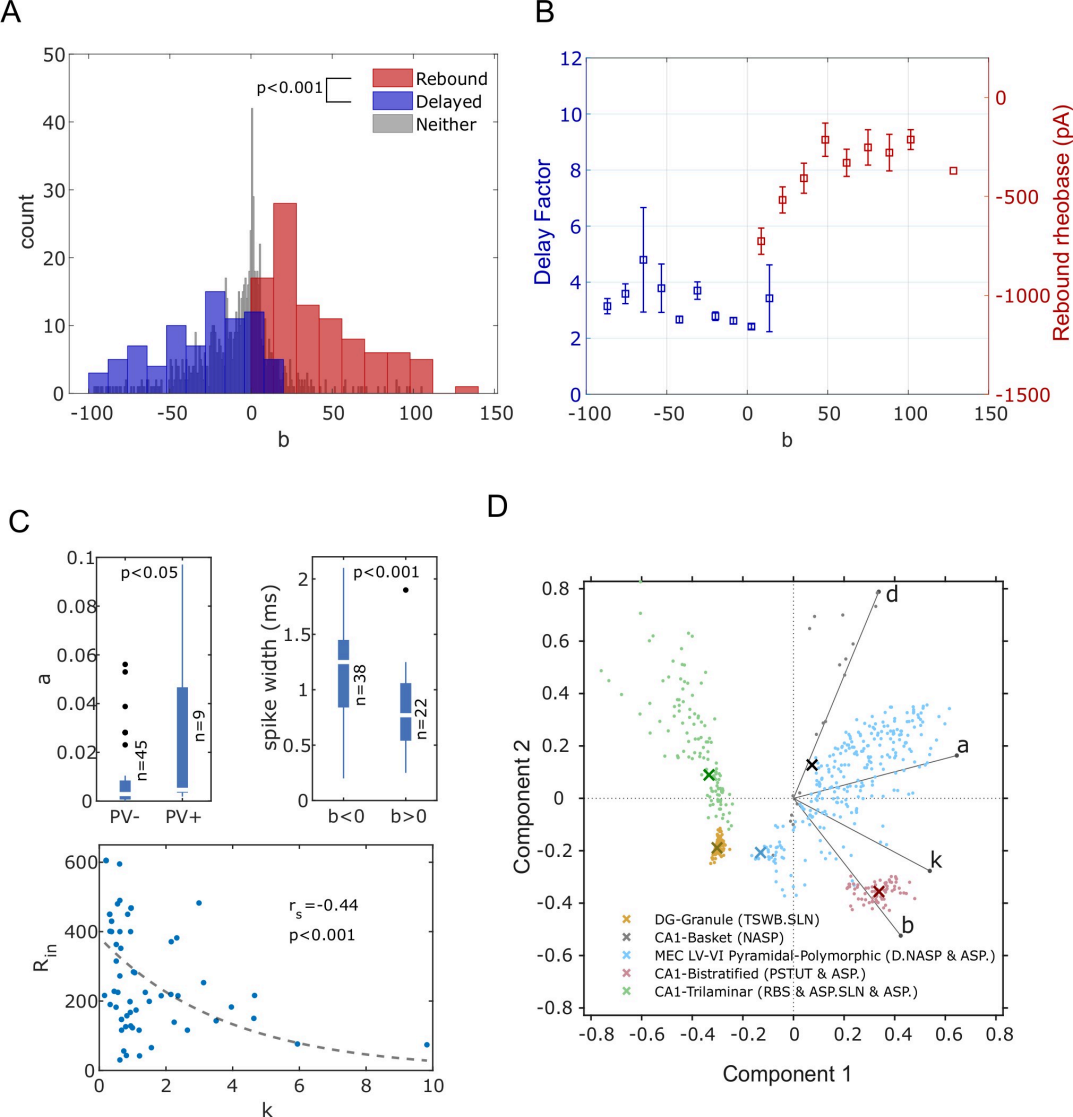
Relationship between model parameters and biological features. (**A**) Distribution of parameter ‘*b*’ for the models that show rebound spiking (red), delayed spiking (blue), and neither (grey). Rebound spiking types were sharply restricted to the positive region of ‘*b*.’ In contrast, delayed spiking types were mostly found in the negative region (two-sample *t-*test). (**B**) Mean and SEM of delay factors and rebound rheobases of the models from the blue and red histogram bins respectively from A. Delay factor is the ratio between *fsl* and average of the first two *ISIs*. Rebound rheobase is the minimum magnitude hyperpolarizing current required to elicit rebound spikes. (**C**) Neuron types with positive expression of Parvalbumin (PV) have higher values for parameter ‘*a*’ than the types with negative expression of PV (one-tailed Wilcoxon Rank-Sum test; p<0.0005 in Anderson-Darling normality test) (top-left). Neuron types with negative values for ‘*b*’ have wider spikes compared to the types with positive values for ‘*b*’ (one-tailed Student *t-*test) (top-right). Input resistance (*R*_*in*_) of a neuron type is negatively correlated with the parameter ‘*k*’ (‘*r*_*s*_’ denotes Spearman’s Rank-Order correlation coefficient, n = 67. An exponential fit was added for illustration only, and not used to measure correlation) (bottom). (**D**) Separation of spike pattern phenotypes in the space of first two principal components. ‘x’ denotes the best model for each type. Vectors denote the principal component coefficients of the respective parameters.

In general, the parameters of the IM collectively determine its spike pattern phenotype. However, the parameter ‘*b*’, which determines if the model is an integrator (*b*<0) or a resonator (*b*>0), sufficiently distinguishes two families of phenotypes. Most of the models that show delayed spiking near their depolarizing rheobases were found in the negative regions of ‘*b*,’ whereas models that show rebound spiking for hyperpolarized input currents were sharply restricted to the positive regions (Fig 8A). These results are consistent with the fact that ‘*b*>0’ is a necessary condition for rebound spiking [17], and we find that most delayed spikers are integrators with the exception of the ones found in the narrow range 0<*b*<20. Thus, rebound (Fig 1C) and delayed (Fig 1D) spiking are, in general, instances of two qualitatively distinct types of intrinsic dynamics.

Next, we studied how much the parameter ‘*b*’ quantitatively influences the respective features in delayed and rebound spiking types. We used delay factors for the former and measured rebound rheobases for the latter. Increasing ‘*b*’ makes the model more rebound excitable until ‘*b’* reached a value of +50, beyond which there was no noticeable effect (Fig 8B). Furthermore, there was no clear trend in the relationship between ‘*b*’ and delay factors. Thus, while ‘*b*’ alone can define a sharp qualitative change in the intrinsic dynamics, its interaction with other parameters, such as ‘*a*,’ determine precise quantitative features. In addition, pairwise correlations revealed several interesting trends between model parameters and electrophysiological or molecular properties of neuron types (Box 1) (also see Fig 8C for the data distributions and further analyses).

**Box 1. Categorical correlations between model parameters and electrophysiological and molecular properties in hippocampal neurons**

1. None of the neuron types that show **PSTUT** has a low value for *‘****k****’* (p<0.01, n = 43). In contrast, none of the neuron types that show **ASP.NASP** has a high value for *‘****k****’* (p<0.05, n = 43). Moreover, no neuron type with high input resistance (***R***_*in*_) has a high value for *‘****k****’* (p<0.001, n = 15) (also see Fig 8C).
2. None of the 23 neuron types except CA3 Lucidum ORAX has **narrow spikes** and a low value for *‘****a****’* (p<0.05). Moreover, clearly positive expressions of somatostatin (**SOM**) tend to co-occur with high values of *‘****a****’* (p<0.05, n = 18).
3. Neuron types with **wide spikes** tend to have negative values for *‘****b****’* (p<0.001, n = 31) (also see Fig 8C).
4. Low values of resting voltage (***V***_***rest***_) tend to co-occur with high values of ‘***d***’ (p<0.05, n = 21). In contrast, no neuron type with positive expression of serotonin (**5HT-3**) has a high value for ‘***d***’ (p<0.05, n = 14).

The p values and sample sizes (n) pertain to Barnard’s exact test for 2 x 2 contingency tables (see ‘Methods’).

Parvalbumin (PV)-positive and somatostatin (SOM)-positive interneurons have been shown to elicit narrow spikes of high frequency, with no or weak spike frequency adaptation [54]. Lower (and higher) values of ‘*a*’ result in slower (and faster) recovery of adaptation variable ‘*U*’ [6], resulting in ASP. (and NASP) behaviors as we reported previously [13]. Consistent with the above facts, high values of ‘*a*’ co-occurred with positive expressions of PV (Fig 8C) and SOM (Box 1), and low values of ‘*a*,’ with one exception, never co-occurred with narrow spikes (Box 1).

Negative values of ‘b’ correspond to a saddle-node bifurcation, which is necessary for the neuron to elicit low-frequency spikes [17]. Our analysis linked negative values of ‘b’ with wide spikes, a well-known property of regular (non-fast) spiking neurons. It should be noted that our modeling approach only enforces the temporal features of the overall spike patterns, such as *sfa*, and not the characteristics of individual spikes, yet makes expected links between the model parameters and spike width in the original (experimental) traces.

Cortical interneurons expressing serotonin receptor 3 (5HT-3) have been shown to elicit spike patterns with sustained *sfa* [55]. The parameter ‘*d*’ is the magnitude of offset for the state variable ‘*U*’ during a spike reset, and high values of ‘*d*’ prevent the model from exhibiting sustained *sfa*. As mentioned before, low values of ‘*a*’ can result in sustained *sfa* (ASP.) behavior. However, the model would quickly reach the steady-state when a low value of ‘*a*’ is combined with a high value of ‘*d*,’ typically resulting in a RASP.NASP pattern. Consistently, high values of ‘*d*’ never co-occurred with expressions of 5HT-3.

Parameter ‘*k*’ scales the difference between the membrane potential state variable (*V*) and threshold voltage (*V*_*t*_) (see eqn-1). Near resting equilibrium (*V*_*r*_), higher values of ‘*k*’ will require higher values of depolarizing ‘*I’* to compensate for the scaling effect to initiate a spike. Thus, ‘*k*’ affects the excitability in the IM (S5 Fig), and higher values of ‘*k*’ result in lower excitability. It is known that neurons with higher rheobase (lower excitability) have lower input resistances (see for instance [56]). Consistent with these facts, high input resistances were never found with high values of ‘*k*.’

Another interesting correlation is that the PSTUT phenotypes were never found for low values of ‘*k*’ (also see S5 Fig). Our analysis revealed that the number of periodic loops in the limit cycle attractor changes from infinite-period (aperiodic/chaotic) to single-period, as the value of ‘*k*’ is decreased in PSTUT phenotypes (not shown in figure). Such higher-periodic attractor loops are necessary to capture the criteria for bursting/stuttering phenotypes in the IM. This suggests a mathematically interesting relationship between ‘*k*’ and the maximum intrinsic periodicity in the model. However, the in-depth study required to fully uncover this relationship is beyond the scope of this study. Nevertheless, these correlations are also consistent with our previous finding that PSTUT neurons never have high input resistances [16] (see S6 Fig for a graphical summary of all the correlations).

Our modeling framework represents each neuron type as a cloud of possibilities in the model parameter space (Fig 8D). Spike patterns produced by all the models in a cloud strictly adhere to the criteria for the respective target qualitative class, but small errors in the quantitative features were accepted to allow variabilities in the spike pattern features (not shown here; see [13] for details on the optimization framework design that allows such variabilities and for examples of ranges of quantitative features). This provides a sampling region for each neuron type to create network models with intragroup neuron variabilities. Several factors affect the size and shape of these clouds: bursting/stuttering phenotypes typically result in smaller clouds than spiking phenotypes. Similarly, spike patterns with unknown input currents (see Fig 1D) result in bigger clouds, since the models are searched in a broad range of input currents for such cases. At present, these clouds are only identified for single-compartment models due to the computational cost of exploring higher dimensional parameter spaces of multi-compartment models.

## Discussion

Hippocampal neurons show diverse features in their morphological, electrical and molecular properties [57]. *Hippocampome.org* (v.1.4) identifies 122 types of neurons defined primarily based on their neurite invasion patterns in the hippocampal parcels [15]. Their intrinsic spike pattern features were extracted from relevant publications, and systematic characterization of such features revealed diverse and complex spike pattern phenotypes among the 122 morphological types [16]. The present work described a comprehensive set of simple models that are accurate quantitative representations of such spike pattern phenotypes. Correlation analysis revealed novel insights into the relationship between IM parameters and various biological features. In addition, point neuron models were compactly extended to create multi-compartment models with up to four compartments. Our compact-MC models, in addition to quantitatively reproducing the somatic spike pattern phenotypes, exhibited voltage attenuation profiles and interplay between different compartments that are consistent with experimental observations and simulations using morpho-MC models (Figs 5 and S3).

The compact-MC models constitute a useful balance between computational efficiency and biological interpretability, but it is also important to recognize their limitations. The distal apical dendrites in CA1 Pyramidal neurons and their morpho-MC models show activity-dependent attenuation of back-propagating spikes, such that the last spike in a spike train attenuates more markedly than the first spike [46,49]. This is due to the activity-dependent changes in the ratio of sodium to potassium current in the dendrites [58]. Our compact-MC models did not show such activity-dependent attenuation of back-propagating spikes. The instantaneous reset of the voltage following its peak in the IM might be a limiting factor in this context. In addition, due to the lack of specific dendritic recordings and higher computational costs of optimizing the compact-MC models, we only enforced a minimum set of general constraints to create qualitatively accurate dendritic compartments. While we lack data to verify if the dendritic compartments in many of our models quantitatively reproduce the properties of their biological counterparts, their somatic compartments are quantitatively accurate (Fig 3). It should also be mentioned that we only considered coupling topologies with consecutive compartments for deeper layers, whereas, in real neurons, the deeper layer synapses may be on different branches than superficial layers. However, our compact-MC representations are useful for studies that aim to extend a baseline network of single-compartment neurons with minimum necessary dendritic properties to enable layer-level connectivity specifications.

Our single-compartment and compact-MC representations emphasize biological realism in the context of somatic intrinsic diversity, but it is also worth discussing their biological realism in the context of intra-neuron type variability in intrinsic dynamics. The intrinsic property of a neuron revealed in its spike patterns is determined by the types and precise distribution of the underlying ion channel conductances, such as sodium, potassium, and calcium. However, it has been shown that similar dynamics can arise from a broad range of combinations of these conductances [10,59–62]. Consistent with this notion, our modeling framework represents a spike pattern phenotype as a cloud of possibilities in the parameter space (Fig 8D). Two closely related considerations motivate such representation.

The first issue is the existence of intrinsic variabilities in the spike pattern features among different neurons of the same type. For example, all the models representing the CA1 Trilaminar type (Fig 8D) were obtained using the features of voltage traces recorded from a single neuron (Fig 1C). While this particular neuron elicited 22 spikes with a *sfa* magnitude of 0.038 for 0.05*nA*, a different CA1 Trilaminar neuron might show slightly different values for these features, under the same input conditions, due to intrinsic variability. Furthermore, the recorded intrinsic spike pattern features might be influenced by the conditions such as the type of recording electrode and difference in animal strain, sex, or age. However, current knowledge about the intrinsic dynamics of these neuron types is limited to the representative traces that the researchers who studied these neuron types chose to publish. Therefore, we allowed a small range in the spike pattern features of a model as long as these features strictly adhere to the definitions of the respective target qualitative class. While the cloud boundaries defining such ranges are currently arbitrary, one could easily enhance our modeling framework to include more realistic ranges, when such ranges are experimentally obtained for all neuron types.

Secondly, neurons have intrinsic plasticity and undergo homeostatic regulations to maintain some constancy in the network activity [61,63–66]. In cell cultures, intrinsic homeostasis has been shown to modify non-synaptic ion channel conductances of pharmacologically isolated neurons. Such modifications shift the input-dependency of a neuron’s responses based on the history of activity. For example, activity deprived neurons showed higher firing rates than control group for the current injections of the same magnitude [66]. In another study, chronic isolation from normal inputs switched a neuron’s response from tonic spiking to intrinsic bursting and this transition was reversed by applying a rhythmic inhibitory drive [63]. While these results suggest that each neuron has a working range that flexibly defines its input-dependent responses, such ranges likely preserve the overall qualitative spike pattern phenotypes [65]. Our EA search for a cloud of models not only included the space of intrinsic IM parameters that define a phenotype, but also included a small range for input current (a 20*pA* range symmetrically encompassing experimental input current magnitude), allowing a reasonable flexibility for its input-dependency.

Considering the issues discussed above, an approach to modeling biological circuits should assume a flexible range for its components. While Hebbian plasticity rules can enable flexible ranges in synaptic conductances, the rules governing a neuron’s intrinsic plasticity remain largely unknown. Although cell-autonomous regulatory rules have been proposed [67], from a network perspective, intrinsic homeostasis has been shown to synergistically result from multiple interacting components in a circuit [68,69]. Exhaustively reductionist approaches to modeling brain regions specify *precise* descriptions at the level of ion channel conductances. While data gathered from different experimental conditions or inevitably from different animals drive such intrinsic descriptions, there is no guarantee that they specify dynamically compatible critical ranges necessary for a higher-level integrative property [70].

A large-scale approach to modeling a brain region, rather than being purely reductionist, should attempt to complement the descriptions of individual components with syntactically relevant descriptions at an integrative level. For example, temporal sequences of activity in ensembles of hippocampal neurons are correlated with the locations of an animal during spatial navigation [71–73]. Such self-organizing ensembles of neurons, in general, have been suggested to form neural syntax [74]. Complex periodic structures in these ensembles, such as theta-modulated gamma activity patterns, should be enforced in a network model as sparse higher-level descriptions.

Future studies should aim to identify a family of models for an experimentally known network-level property, within the anatomical constraints of connectivity among hippocampal neuron types [75], using the sampling regions for those types created in this study. Then, the identified family of models should be evaluated for their predictive power, or one could investigate how the predictive abilities increase by scaling up the network or by adding more mechanisms, such as synaptic plasticity and spatial context for synaptic integration. This approach emphasizes the goal of creating the simplest model with the most predictive power iteratively.

Finally, it is important to identify recurring patterns of self-organization in biological complex systems and translate such patterns into mathematical descriptions that could be enforced using optimization techniques, such as an EA that heuristically explores the given parameter space. If a biological complex system can indeed allow flexibility and compensation among multi-level components, then it suggests that a certain property could emerge from multiple, similar configurations in a network parameter space, which a metaheuristic approach [76], such as an EA, can take advantage of. While this might be a computationally expensive task, our simple models, with only two state variables per neuron, as opposed to hundreds in a biophysically detailed multi-compartment model, allow one to approach this problem much more efficiently. Future releases of *Hippocampome.org* are aimed at approximating the counts of different neuron types and mapping synaptic properties to potential connections. These enhancements will further narrow down the space of biological possibilities to create realistic large-scale models of hippocampal circuits.

## Supporting information

S1 Text Identifying firing pattern phenotypes of hippocampal neuron types. (PDF)

S1 Fig Models reproduce frequency-response curves of different neuron types. Experimentally measured mean firing frequencies of (**A**) CA1 Pyramidal [77], (**B**) DG HICAP [25] and (**C**) DG Granule [78] for various input current magnitudes (±5pA), were used to constrain the model responses. Representative experimental (black) and corresponding model (red) traces are given in the bottom. Calibrations: 20mV, 100ms (left); 20mV, 200ms (middle); 40mV, 40ms (right).
(PDF)

S2 Fig Multi-compartment models capture qualitative dendritic properties and sub- and supra-threshold signal propagation. (A) Four different layouts of asymmetrically coupled compartments from Fig 3. (B) Minimum depolarizing input required to elicit a spike (I-rheo) and steady-state voltage deflection (V-def) for a hyperpolarizing input are higher in dendritic-compartments than in the somatic-compartment. (C) A single synapse stimulated at a dendritic-compartment (denoted by ‘*’) evokes a unitary EPSP at the somatic-compartment (red traces), with an amplitude in the range [0.1, 0.9] mV. (D) Coupling mechanism implemented in the models allows forward propagation of spikes initiated at a dendritic-compartment (denoted by ‘*’).
(PDF)

S3 Fig Dendritic properties of the 3-compartment DG Granule model. Stratum Granulosum (SG), Stratum moleculare-inner (SMi), and Stratum moleculare-outer (SMo) denote the somatic, proximal, and distal dendritic compartments, respectively. (**A)** Attenuation of first back-propagating action potential (AP) from experimental recordings (exp.), biophysically and morphologically detailed multi-compartment model (morpho-MC) and three-compartment IM. Inset shows compartment responses for somatic current injection. (**B)** Attenuation of dendritic EPSPs as they propagate towards soma. Inset shows compartment responses for a single synaptic stimulation at SMi and SMo. Experimental and model data were digitized from [23]. Calibrations: 25mV, 20ms (A) and 2mV (B).
(PDF)

S4 Fig Additional compartments are necessary to capture the complex transient pattern ‘RASP.ASP.’. (A) Experimentally recorded voltage trace from a CA1 Basket CCK+ neuron [79] digitized by *Hippocampome.org*. (B) 4-compartment model reproduces the pattern RASP.ASP. (red), and the single-compartment counterpart failed to do so (blue). (C) While both versions reproduce nISIs accurately, the multi-compartment model more accurately reproduces sfa. Sfa-1 is the rapid frequency adaptation measured in the first three ISIs (RASP.), and sfa-2 is the weak adaptation measured in the remaining 35 ISIs (ASP.) Note that sfa-2 = 0 in the single-compartment model. Spike amplitudes are truncated.
(PDF)

S5 Fig Parameter ‘k’ affects the excitability level in the IM. Arrows represent the CA1 Bistratified model (see Fig 2). Model responses were classified by only varying the parameter ‘*k*’ and input current ‘*I*’ for this plot. As the value of ‘*k*’ is increased, higher values of depolarizing current are required to elicit stuttering (yellow) or spiking (light green) patterns. Notice that the stuttering behavior occurs just above the rheobase and is non-existent for ‘*k’*<1.75. It should also be noted that the stuttering region could be wider in a different sub-region of the parameter space.
(PDF)

S6 Fig A graphical summary of the correlations between model parameters and biological properties of neuron types. Green and red indicate the presence and absence, respectively, of a feature (see Box 1 and Fig 8C for details).
(PDF)

S1 Table Abbreviations. (A) Quantitative features of firing patterns. (B) Elements of firing patterns. (C) Other abbreviations.
(PDF)

S2 Table Features and errors for all the traces from Fig 1. (PDF)

S3 Table Features and errors for all the traces from Fig 2. (A) Errors in spiking features. (B) Errors in bursting/stuttering features.
(PDF)

S4 Table Features and errors for all the traces from Fig 3. (A) Errors in spiking features. (B) Errors in bursting/stuttering features.
(PDF)
