# Supplementary material for: Simple models of quantitative firing phenotypes in hippocampal neurons: Comprehensive coverage of intrinsic diversity
Source: PLoS Comput Biol. 2019 Oct 28;15(10):e1007462. doi: 10.1371/journal.pcbi.1007462 (PMC6837624; doi:10.1371/journal.pcbi.1007462)
Supplement: S1 Text — (PDF) [file pcbi.1007462.s001.pdf]

S1 Text. Identifying firing pattern phenotypes of hippocampal neuron types

Note: This section summarizes the firing pattern identification scheme from [16], which was under review at the time this section was written.

All analyzed firing pattern traces were recorded in slice preparations, of which 74% were obtained using whole-cell patch clamp and the remaining were obtained using sharp-microelectrodes. The recording system and other important metadata affecting firing (animal species, strain, and age; slice orientation and thickness; intra- and extra-cellular solution compositions, and temperature) are individually annotated for each firing pattern trace at Hippocampome.org [16]. Temporal features of a firing pattern such as *fsl*, *pss*, and *ISIs* (see supplementary table S1A) were used to identify various firing pattern elements (see supplementary table S1B) as follows: A spike pattern includes the element "D," if its *fsl* is at least more than twice the average of the first two *ISIs*. Similarly, a spike pattern with "SLN" has a *pss* that is at least more than twice the average of the last two *ISIs*. Furthermore, *pss* should be more than twice the maximum *ISI* in the pattern. A pattern includes "TSTUT," if there is an inter-spike interval (ISI_i) present within the first five spikes, such that ISI_i is more than $2.5 \times ISI_{i-1}$ and $1.5 \times ISI_{i+1}$. Moreover, the average of all the intervals that follow ISI_i ($ISI_{i+1:n}$, where n is the total number of ISIs) should be at least more than 2.5 times the average of all the intervals that precede it ($ISI_{1:\,i-1}$), and $ISI_{1:\,i-1}$ should have a minimum frequency of 25*Hz*. These protocols check for the presence of a sufficiently longer interval that separates a transient high-frequency cluster of spikes from the rest of the spike train to qualify it as "TSTUT."

The following steps are carried out to identify various subclasses of frequency-adapting spike patterns and their steady states: piecewise linear regression with increasing number of parameters is performed on normalized *ISIs* plotted against their latencies. A regression model with a certain number of parameters is accepted only if it statistically significantly improves the accuracy of a model with less parameters. For example, a spike pattern without frequency adaptation (i.e. *ISI* is constant over time) only requires one parameter ($Y = C$, where C is the Y-intercept of the linear fit) and is identified as "NASP". However, a pattern with frequency adaptation requires two or more parameters. A pattern is identified as "ASP." only if a two-parameter fit ($Y = mX + C$, where m is the slope, and C is the Y-intercept) significantly improves the accuracy over the one-parameter fit ($Y = C$). Similarly, a pattern is identified as "ASP.NASP" only if a three-parameter fit ($Y_1 = m_1X_1 + C_1$, $Y_2 = C_2$, where C_2 accounts for the non-adapting spiking that follows the transient adapting spiking) significantly improves the accuracy over the two-parameter fit ($Y = mX + C$). Similarly, "ASP.ASP." is a pattern, where a four-parameter fit

was significantly better than the three-parameter fit. Furthermore, if a strong frequency adaptation is only present in the first 2 or 3 *ISIs*, it is marked as “RASP.” The slopes and Y-intercepts of the piecewise linear fit quantifies the feature *sfa*.

A pattern is identified to have the element “PSTUT,” if the sum of ratios of maximum ISI (candidate for inter-burst interval) and the preceding *ISI*, and maximum *ISI* and the following *ISI* is at least more than 5. The elements “TSWB” and “PSWB” follow the same protocols as “TSTUT” and “PSTUT,” respectively, except that they indicate the presence of a slow after-hyperpolarizing wave with an amplitude >5mV. Finally, a spike pattern that is elicited after the offset of a hyperpolarizing step input current is labeled as “RBS.”

Thus, a firing pattern can consist of one or more elements, which are separated by dots (‘.’). For example, “D.RASP.NASP” is a 3-element pattern that shows a strong *sfa* only in the first 2 or 3 ISIs following a long *fsl* and followed by a non-adapting spike train. This pattern has two transient elements (“D” and “RASP”) and one steady-state element (NASP). The set of all firing patterns observed from a neuron type forms its firing pattern phenotype. A phenotype belongs to stuttering/bursting super-family, if at least one firing pattern consists of TSTUT/TSWB or PSTUT/PSWB. Otherwise, the phenotype belongs to the spiking super-family.
