# Supplementary figures and images for: Simple models of quantitative firing phenotypes in hippocampal neurons: Comprehensive coverage of intrinsic diversity
Source: PLoS Comput Biol. 2019 Oct 28;15(10):e1007462. doi: 10.1371/journal.pcbi.1007462 (PMC6837624; doi:10.1371/journal.pcbi.1007462)

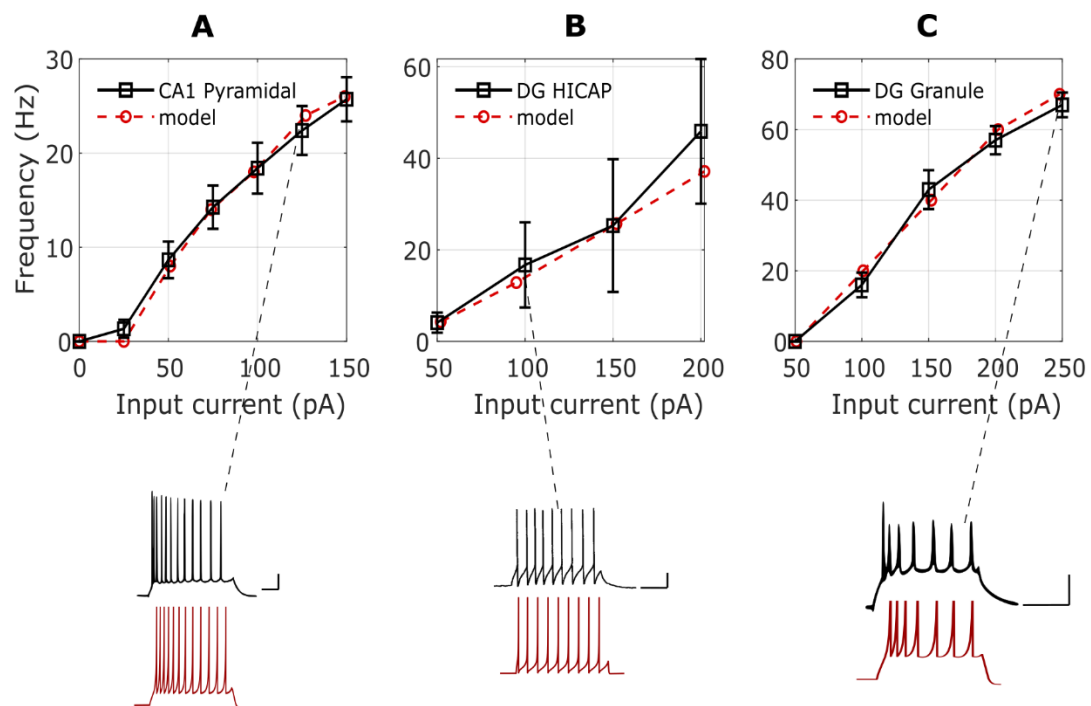

Supplement: S1 Fig — Experimentally measured mean firing frequencies of (A) CA1 Pyramidal [77], (B) DG HICAP [25] and (C) DG Granule [78] for various input current magnitudes (±5pA), were used to constrain the model responses. Representative experimental (black) and corresponding model (red) traces are given in the bottom. Calibrations: 20mV, 100ms (left); 20mV, 200ms (middle); 40mV, 40ms (right). (PDF) [file pcbi.1007462.s002.pdf]

**A**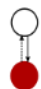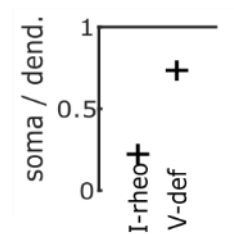**C**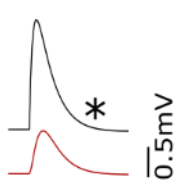**D**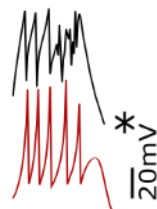**B**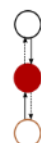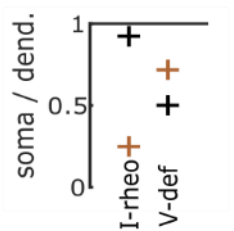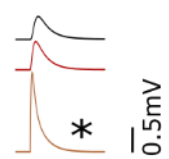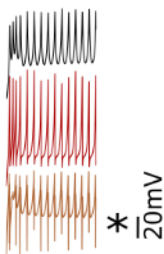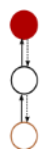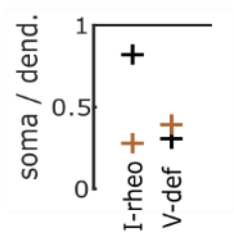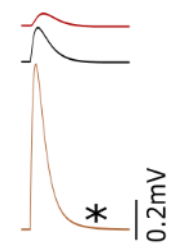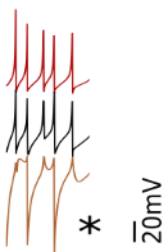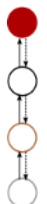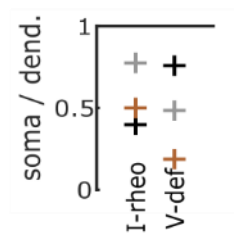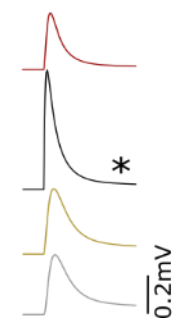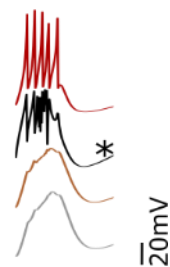

Supplement: S2 Fig — (A) Four different layouts of asymmetrically coupled compartments from Fig 3. (B) Minimum depolarizing input required to elicit a spike (I-rheo) and steady-state voltage deflection (V-def) for a hyperpolarizing input are higher in dendritic-compartments than in the somatic-compartment. (C) A single synapse stimulated at a dendritic-compartment (denoted by ‘*’) evokes a unitary EPSP at the somatic-compartment (red traces), with an amplitude in the range [0.1, 0.9] mV. (D) Coupling mechanism implemented in the models allows forward propagation of spikes initiated at a dendritic-compartment (denoted by ‘*’). (PDF) [file pcbi.1007462.s003.pdf]

**A**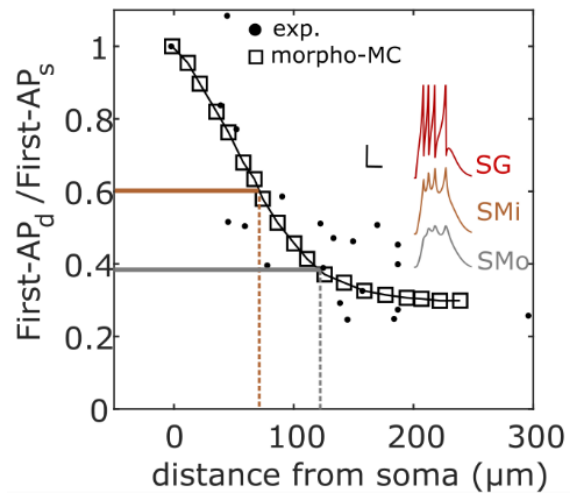**B**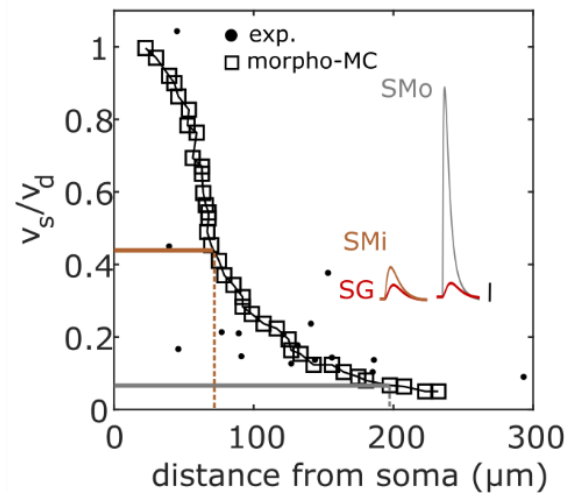

Supplement: S3 Fig — Stratum Granulosum (SG), Stratum moleculare-inner (SMi), and Stratum moleculare-outer (SMo) denote the somatic, proximal, and distal dendritic compartments, respectively. (A) Attenuation of first back-propagating action potential (AP) from experimental recordings (exp.), biophysically and morphologically detailed multi-compartment model (morpho-MC) and three-compartment IM. Inset shows compartment responses for somatic current injection. (B) Attenuation of dendritic EPSPs as they propagate towards soma. Inset shows compartment responses for a single synaptic stimulation at SMi and SMo. Experimental and model data were digitized from [23]. Calibrations: 25mV, 20ms (A) and 2mV (B). (PDF) [file pcbi.1007462.s004.pdf]

**A**

CA1 Basket CCK+

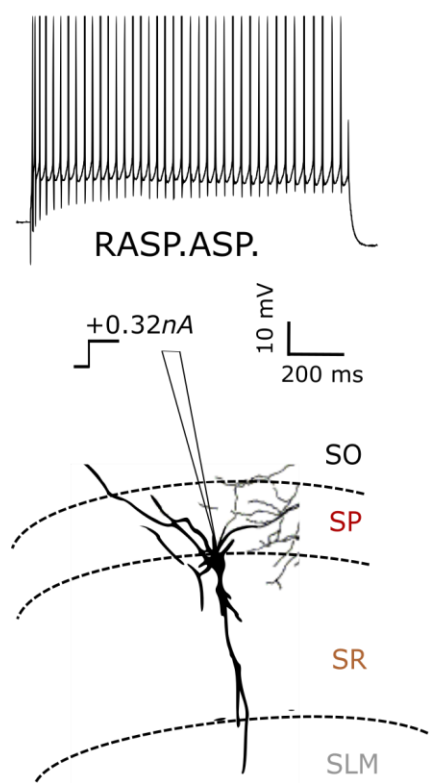**B**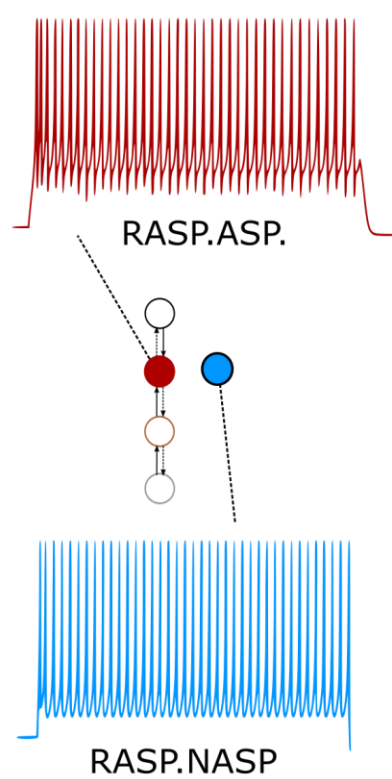**C**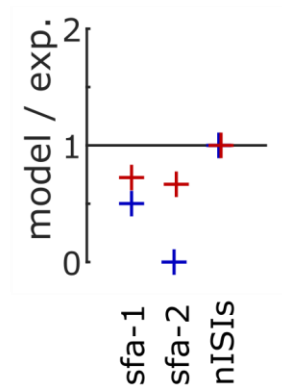

Supplement: S4 Fig — (A) Experimentally recorded voltage trace from a CA1 Basket CCK+ neuron [79] digitized by Hippocampome.org. (B) 4-compartment model reproduces the pattern RASP.ASP. (red), and the single-compartment counterpart failed to do so (blue). (C) While both versions reproduce nISIs accurately, the multi-compartment model more accurately reproduces sfa. Sfa-1 is the rapid frequency adaptation measured in the first three ISIs (RASP.), and sfa-2 is the weak adaptation measured in the remaining 35 ISIs (ASP.) Note that sfa-2 = 0 in the single-compartment model. Spike amplitudes are truncated. (PDF) [file pcbi.1007462.s005.pdf]

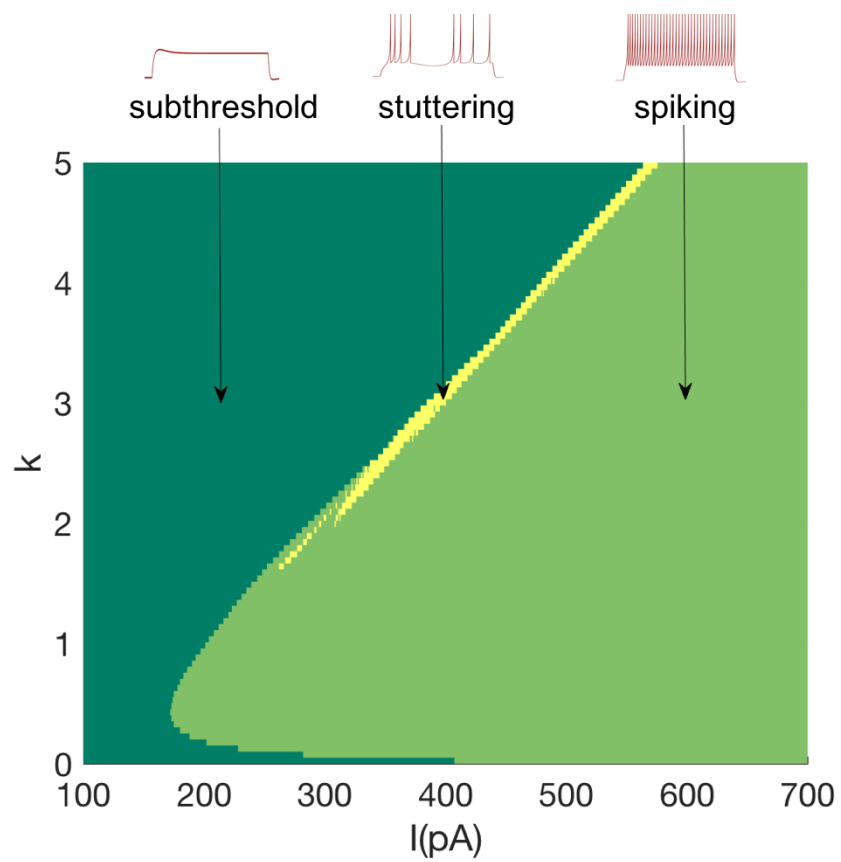

Supplement: S5 Fig — Arrows represent the CA1 Bistratified model (see Fig 2). Model responses were classified by only varying the parameter ‘k’ and input current ‘I’ for this plot. As the value of ‘k’ is increased, higher values of depolarizing current are required to elicit stuttering (yellow) or spiking (light green) patterns. Notice that the stuttering behavior occurs just above the rheobase and is non-existent for ‘k’<1.75. It should also be noted that the stuttering region could be wider in a different sub-region of the parameter space. (PDF) [file pcbi.1007462.s006.pdf]

*high Rin*  
*ASP.NASP*

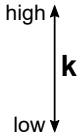

*PSTUT*

*SOM+*  
*PV+*

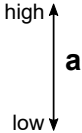

*narrow spikes*

*5HT-3+*  
*low Vrest*

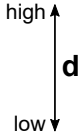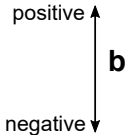

*wide spikes*

Supplement: S6 Fig — Green and red indicate the presence and absence, respectively, of a feature (see Box 1 and Fig 8C for details). (PDF) [file pcbi.1007462.s007.pdf]
