# Supplementary material for: Simple models of quantitative firing phenotypes in hippocampal neurons: Comprehensive coverage of intrinsic diversity
Source: PLoS Comput Biol. 2019 Oct 28;15(10):e1007462. doi: 10.1371/journal.pcbi.1007462 (PMC6837624; doi:10.1371/journal.pcbi.1007462)
Supplement: S1 Table — (A) Quantitative features of firing patterns. (B) Elements of firing patterns. (C) Other abbreviations. (PDF) [file pcbi.1007462.s008.pdf]

S1A Table. Quantitative features of firing patterns

fsl	First Spike Latency
sfa	Spike Frequency Adaptation
ISI	Inter-Spike Interval
nISIs	number of ISIs
pss	Post-Spike Silence
n_bursts	Number of bursts
bw	Burst Width
pbi	Post-Burst Interval
b-nISIs	nISIs within a Burst

S1B Table. Elements of firing patterns

D	Delayed
ASP	Adapting SPiking
NASP	Non-Adapting SPiking
RASP	Rapidly Adapting SPiking
TSTUT	Transient Stuttering
TSWB	Transient Slow Wave Bursting
PSTUT	Persistent Stuttering
PSWB	Persistent Slow Wave Bursting
SLN	SiLeNce
RBS	ReBoundSpiking

S1C Table. Other abbreviations

IM	Izhikevich Model
compact-MC	Compact Multi-Compartment model
morpho-MC	Morphologically detailed Multi-Compartment model
EA	Evolutionary Algorithm
SP	Stratum Pyramidale
SR	Stratum Radiatum
SLM	Stratum Lacunosum Moleculare
SO	Stratum Oriens
SG	Stratum Granulosum
SMi	Inner one-third of Stratum Moleculare
SMo	Outer one-third of Stratum Moleculare
H	Hilus
