# Supplementary material for: Simple models of quantitative firing phenotypes in hippocampal neurons: Comprehensive coverage of intrinsic diversity
Source: PLoS Comput Biol. 2019 Oct 28;15(10):e1007462. doi: 10.1371/journal.pcbi.1007462 (PMC6837624; doi:10.1371/journal.pcbi.1007462)
Supplement: S2 Table — (PDF) [file pcbi.1007462.s009.pdf]

S2 Table. Features and errors for all the traces from Fig 1

Neuron type	pattern class	*I (pA)* exp, model	*fsl (ms)* exp, model, model/exp	*sfa* exp, model, model/exp	*nISIs* exp, model, model/exp	*pss (ms)* exp, model, model/exp
DG Total Molecular Layer	-	50, 42	150, 367, **2.45**	-	1, 1, **1***	-
	ASP.	75, 73	64.71, 96, **1.49**	y=0.142x+0.947, y=0.142x+0.975, **1.01**	5, 5, **1**	99.06, 96.96, **0.98**
	ASP.	100, 102	22.25, 64, **2.88**	y=0.114x+0.911, y=0.082x+0.987, **0.90**	9, 9**, 1**	52.97, 27.37, **0.52**
	ASP.	200, 205	13.05, 33, **2.53**	y=0.056x+1.073, y=0.032x+1.014, **0.76**	19, 19, **1**	18.67, 17.40, **0.93**
CA1 Basket	NASP	150, 151	175.00, 141, **0.81**	y=0.000x+1.197, y=0.000x+1.197, **1**	2, 2, **1**	210, 210.15, **1**
	NASP	310, 316	4.97, 18.00, **3.62**	y=0.000x+2.080, y=0.000x+1.165, **0.56**	55, 55, **1**	20.90, 0, **0**
CA1 Trilaminar	RBS	-100, -100	770, 810**, 1.05**	-	1, 1, **1**	-
	ASP.SLN	25, 23	50.73, 56.00, **1.1**	y=0.720x+1.000, y=0.491x+1.000, **0.84**	2, 2, **1**	541.95, 542.17, **1**
	ASP.	50, 46	14.49, 28, **1.93**	y=0.038x+1.340, y=0.036x+1.006, **0.85**	21, 21, **1**	26.21, 24.59, **0.94**
MEC LV-VI Pyramidal Polymorphic	D.NASP	unknown, 78	292.34, 292, **1**	y=0.000x+1.055, y=0.000x+1.042, **0.99**	5, 5, **1**	40.00, 63.57, **1.59**
	ASP.	200, 197	56.40, 56.00, **0.99**	y=0.036x+1.103, y=0.030x+1.024, **0.88**	18, 18, **1**	23.70, 23.33, **0.98**

*number of spikes is compared for single-spike traces
