# Supplementary material for: Simple models of quantitative firing phenotypes in hippocampal neurons: Comprehensive coverage of intrinsic diversity
Source: PLoS Comput Biol. 2019 Oct 28;15(10):e1007462. doi: 10.1371/journal.pcbi.1007462 (PMC6837624; doi:10.1371/journal.pcbi.1007462)
Supplement: S3 Table — (A) Errors in spiking features. (B) Errors in bursting/stuttering features. (PDF) [file pcbi.1007462.s010.pdf]

S3A Table. Features and errors for all the traces from Fig 2 (spiking features)

Neuron type	pattern class	*I (pA)* exp, model	*fsl (ms)* exp, model, model/exp	*sfa* exp, model, model/exp	*nISIs* exp, model, model/exp	*pss (ms)* exp, model, model/exp
CA1 Bistratified	PSTUT	400, 408	40.48, 37.00, **0.91**	NA	NA	43.48, 43.75, **1.01**
	ASP.	600, 602	12.38, 16.00, **1.29**	y=0.008x+1.213, y=0.005x+1.187, **0.8**	35, 35**, 1**	9.63, 0, **0**
EC LV Deep Pyramidal	TSTUT.NASP	400, 410	16.00, 15.00, **0.94**	NA	NA	164, 126, **0.77**
DG Granule	TSWB.SLN	200, 208	41.81, 13.00, **0.31**	NA	1, 1, **1**	1904.27, 1902.08, **1.0**
	TSWB.SLN	400, 404	7.79, 7.00, **0.9**	NA	3, 4, **1.33**	1943.55, 1943.16, **1.0**
DG Hilar Ectopic Granule	TSWB.SLN	unknown, 602	14.34, 17.00, **1.19**	NA	1, 1, **1**	156.16, 129.95, **0.83**
	ASP.	700, 699	9.52, 10, **1.05**	y=0.791x+0.693, y=0.790x+0.683, **0.99**	2, 2, **1**	107.94, 109.29, **1.01**

S3B Table. Features and errors for all the traces from Fig 2 (bursting/stuttering features)

Neuron type	pattern class	*I (pA)* exp, model	*n_bursts* exp, model, model/exp	*bw* (ms)* exp, model, model/exp	*pbi* (ms)* exp, model, model/exp	*b-nISIs** exp, model, model/exp
CA1 Bistratified	PSTUT	400, 408	2, 2**, 1**	83, 70 56, 69, **1.04**	154, 154**, 1**	3, 3, 2, 2, **1**
EC LV Deep Pyramidal	TSTUT.NASP	400, 410	2, 2, **1**	88, 38 3752, 3654, **0.7**	276, 365**, 1.32**	2, 2, 16, 19, **1.09**

*features are reported for each burst in a pattern
