# Supplementary material for: Simple models of quantitative firing phenotypes in hippocampal neurons: Comprehensive coverage of intrinsic diversity
Source: PLoS Comput Biol. 2019 Oct 28;15(10):e1007462. doi: 10.1371/journal.pcbi.1007462 (PMC6837624; doi:10.1371/journal.pcbi.1007462)
Supplement: S4 Table — (A) Errors in spiking features. (B) Errors in bursting/stuttering features. (PDF) [file pcbi.1007462.s011.pdf]

S4A Table. Features and errors for all the traces from fig 3 (spiking features)

Neuron type	pattern class	*I (pA)* exp, model	*fsl (ms)* exp, model, model/exp	*sfa* exp, model, model/exp	*nISIs* exp, model, model/exp	*pss (ms)* exp, model, model/exp
CA1 Perforant Path Associated	ASP.SLN	700, 708	0.16, 12, **75**	y=0.142x+0.915, y=0.115x+0.852, **0.875**	12, 12, **1**	59.55, 59.10, **0.99**
DG MOLAX	PSTUT	400, 397	15.30, 43, **2.81**	NA	NA	56.90, 0, **0**
MEC LIII Multipolar	RBS	-200, 193	625, 625, **1**	NA	1, 1, **1**	NA
	NASP	250, 243	4.05, 8, **1.98**	y=0.000x+1.310, y=0.000x+1.401, **1.06**	53, 53, **1**	7.37, 0, **0**
DG AIPRIM	ASP.NASP	200, 209	0.45, 14, **31.11**	y1=0.031x1+1.268, y1=0.040x1+1.011; y2=0.000x2+3.576, y2=0.000x2+3.567, **1.02**	33, 33, **1**; 24, 24, **1**	1.24, 13.26, **10.7**
CA1 Pyramidal	ASP.	150, 154	29.16, 89, **3.05**	y=0.085x+1.140, y=0.078x+1.137, **0.96**	11, 11, **1**	120.79, 72.12, **0.6**

S4B Table. Features and errors for all the traces from fig 3 (bursting/stuttering features)

Neuron type	pattern class	*I (pA)* exp, model	*n_bursts* exp, model, model/exp	*bw* (ms)* exp, model, model/exp	*pbi* (ms)* exp, model, model/exp	*b-nISIs** exp, model, model/exp
DG MOLAX	PSTUT	400, 397	7, 6**, 0.86**	39.90, 45; 0, 39; 47.8, 28; 0, 38; 23.4, 29; 24.5, 0, **0.49**	34, 67; 39.2, 95; 66, 65; 62.8, 88; 64.9, 72; 74.4, 0, **1.32**	4, 3; 1, 2; 3, 2; 1, 2; 2, 2; 2, 1, **1.15**

*features are reported for each burst in a pattern
